# Supplementary material for: Macropinocytosis Renders a Subset of Pancreatic Tumor Cells Resistant to mTOR Inhibition
Source: Cell Rep. 2020 Feb 25;30(8):2729–2742.e4. doi: 10.1016/j.celrep.2020.01.080 (PMC7043007; doi:10.1016/j.celrep.2020.01.080)
Supplement: Document S1. Figures S1–S7 and Tables S1 and S2 [file mmc1.pdf]

Cell Reports, Volume *30*

Supplemental Information

Macropinocytosis Renders a Subset of Pancreatic Tumor Cells Resistant to mTOR Inhibition

Evdokia Michalopoulou, Francesca R. Auciello, Vinay Bulusu, David Strachan, Andrew D. Campbell, Jacqueline Tait-Mulder, Saadia A. Karim, Jennifer P. Morton, Owen J. Sansom, and Jurre J. Kamphorst

S1.A

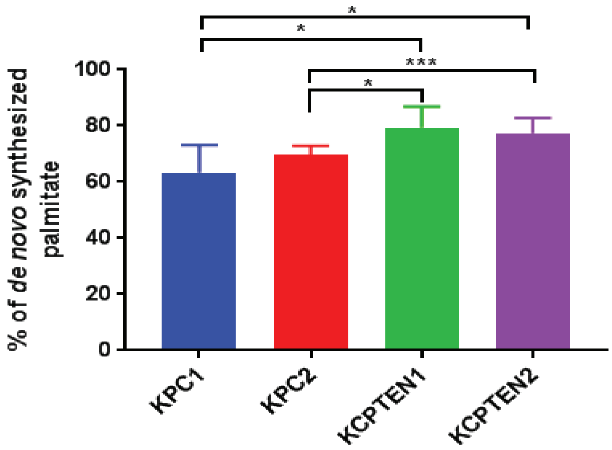

% of *de novo* synthesized palmitate
0
20
40
60
80
100
KPC1
KPC2
KCPTEN1
KCPTEN2
*
*
***
*

S1.B

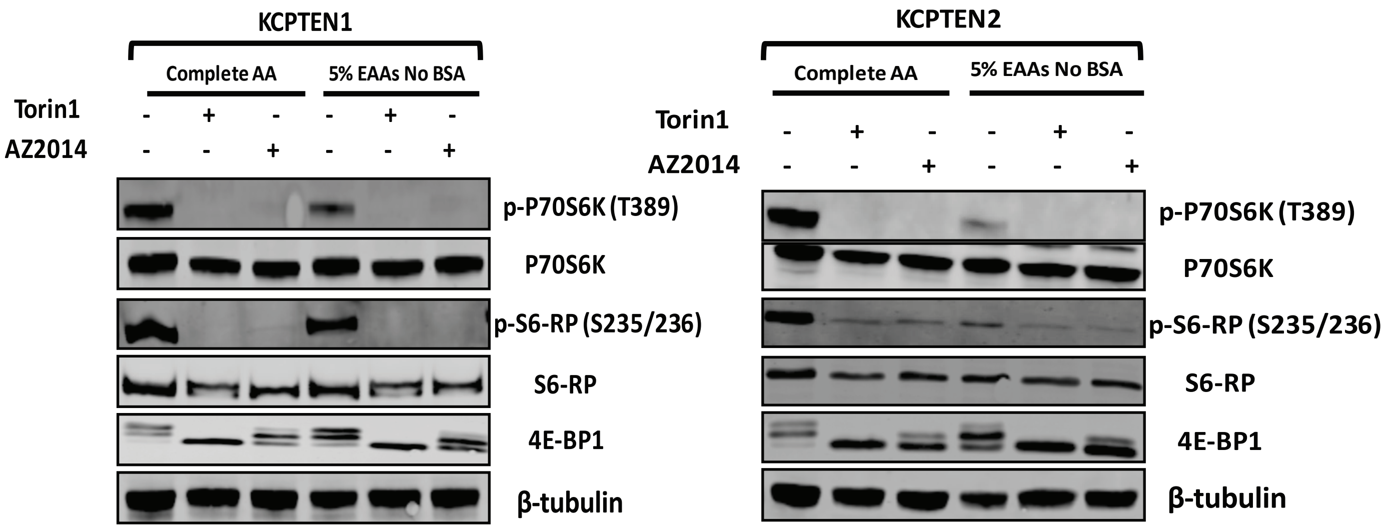

KCPTEN1
Complete AA
5% EAAs No BSA
Torin1
AZ2014
p-P70S6K (T389)
P70S6K
p-S6-RP (S235/236)
S6-RP
4E-BP1
β-tubulin
KCPTEN2
Complete AA
5% EAAs No BSA
Torin1
AZ2014
p-P70S6K (T389)
P70S6K
p-S6-RP (S235/236)
S6-RP
4E-BP1
β-tubulin

S1.C

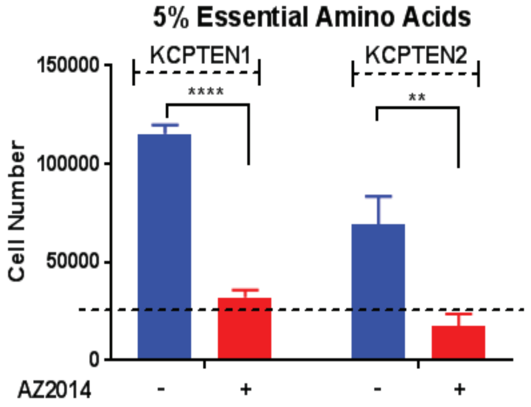

5% Essential Amino Acids
KCPTEN1
KCPTEN2
****
**
Cell Number
0
50000
100000
150000
AZ2014
-
+
-
+

Figure S1. Relate to Figure 1.

KCPTEN cells are sensitive to mTOR inhibition.

- **A** *De novo* synthesized palmitate in KPCs and KCPTENs. Cells were cultured in complete media containing [$U^{13}C$]-glucose and [$U^{13}C$]-glutamine for 72 h.
- **B** mTORC1 signaling activity upon mTOR dual inhibition. Cells were cultured in indicated conditions for 24 h and protein levels of mTORC1 targets were assessed by WB.
- **C** Effect of mTOR dual inhibition (1 µM AZD2014) on proliferation of KC-PTEN cells. Cells were cultured in 5% of DMEM essential amino acid (EAA) concentrations for 72 h.

  For **A**, error bars represent Standard Deviation (SD) of 2 independent experiments each conducted in triplicates and significance was determined by unpaired t-test; for **C**, error bars represent s.e.m. of 3 biological experiments each conducted with 3 technical replicates and significance was determined by Tukey-corrected two-way ANOVA. ****$p<0.0001$, ***$p<0.001$, *$p<0.01$

Dashed line indicates the starting cell number.

S2.A

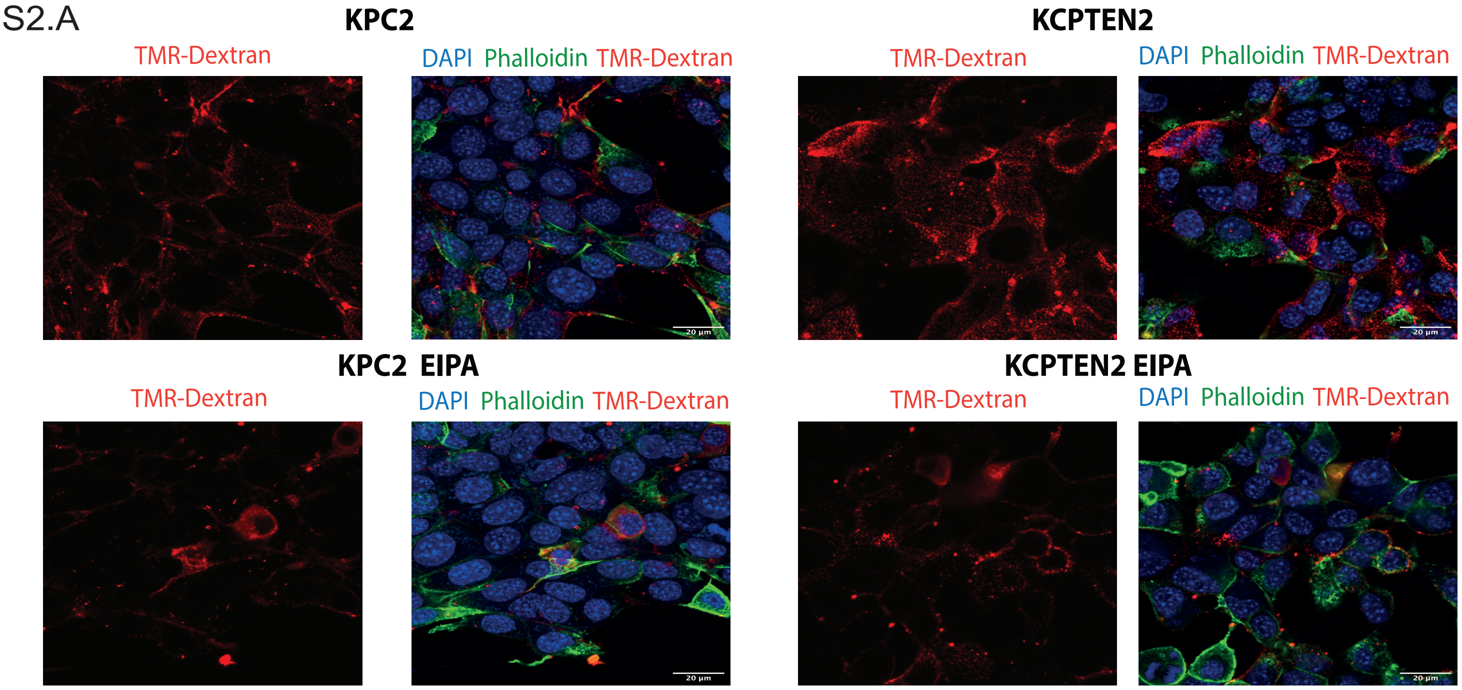

S2.B

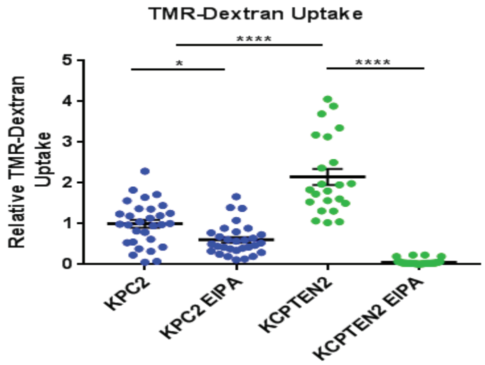

S2.C

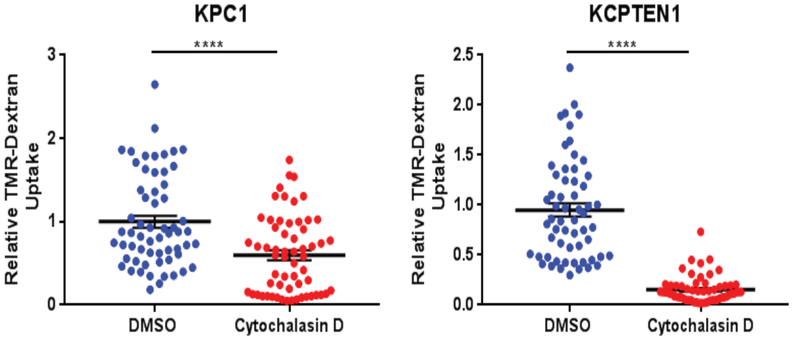

S2.D

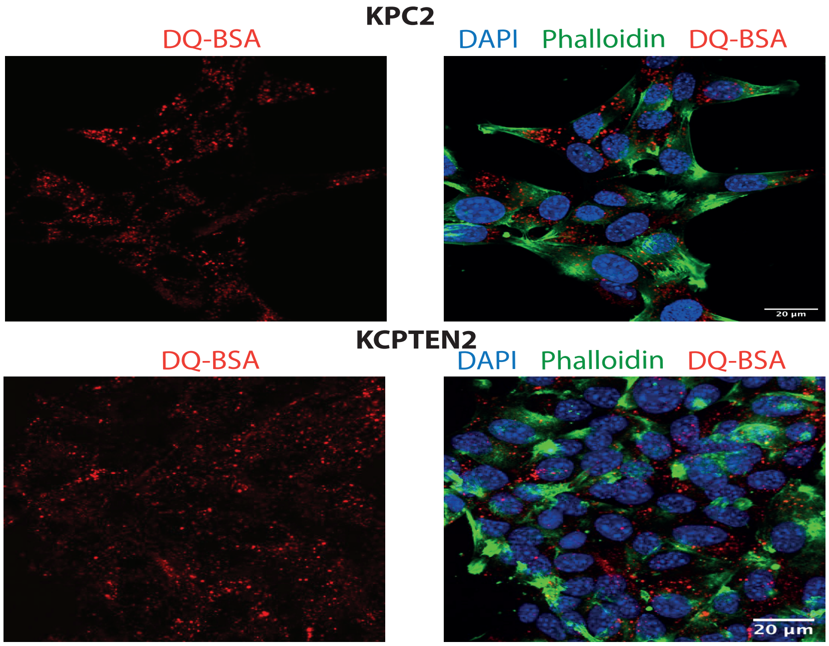

S2.E

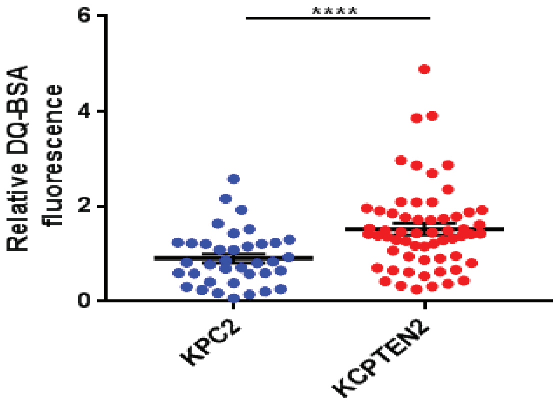

Figure S2. Related to Figure 2.

KCPTEN cells exhibit increased uptake and processing of extracellular material via macropinocytosis.

A Macropinocytosis assay using TMR-Dextran as a marker of macropinosomes (red staining) in KPC and KCPTEN cells. Nuclei stained with DAPI (blue) (49,6-diamidino-2-Phenylindole) and Alexa-488 Phalloidin (green) for actin staining (cellular periphery). The amiloride macropinocytosis inhibitor EIPA was used at 50 µM (1 hour).

B Quantification of TMR-Dextran fluorescence. An average of 30 images was acquired per condition. Values were normalized to the average of untreated KPC2 (DMSO).

C Same as **B**, but 2 µM Cytochalasin D (1 hour) was used to block macropinocytosis.

D Lysosomal processing of extracellular proteins up-taken via macropinocytosis was assessed by DQ-BSA fluorescence.

E Quantification of DQ-BSA fluorescence. An average of 40 z-stack images was acquired per cell line. Values were normalized to the average of KPC2.

For **B, C** & **D**, error bar represents s.e.m. of 2 biological experiments each with 3 technical replicates. For **B** & **D**, significance was determined by one-way ANOVA. For **C,** significance was determined by unpaired student's t-test with Welch's corrections. ns, non-significant. ****$p<0.0001$, *$p<0.05$.

S3.A

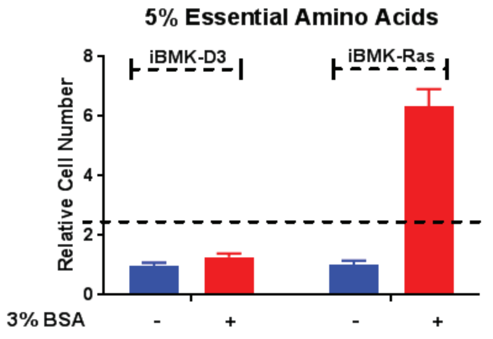

S3.B

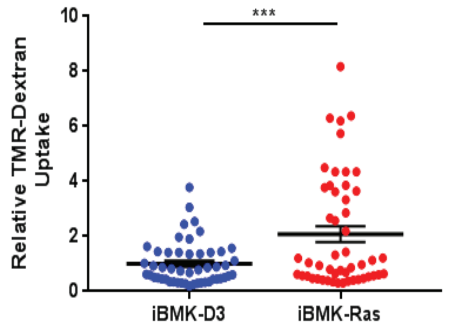

S3.C

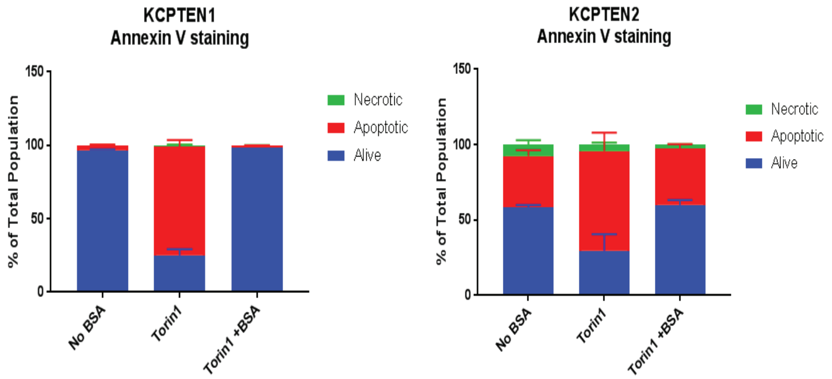

S3.D

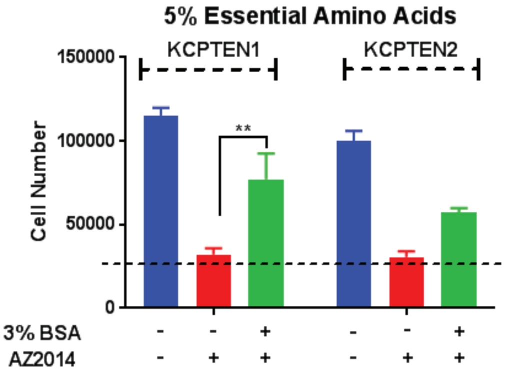

S3.E

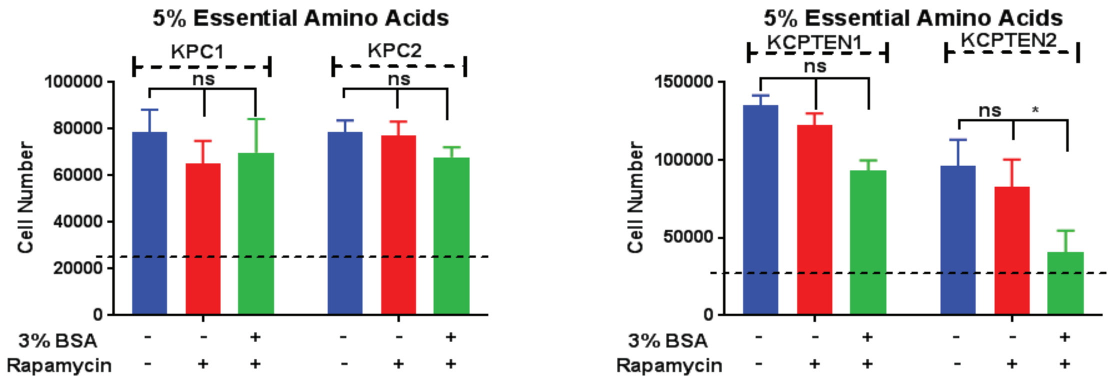

S3.F

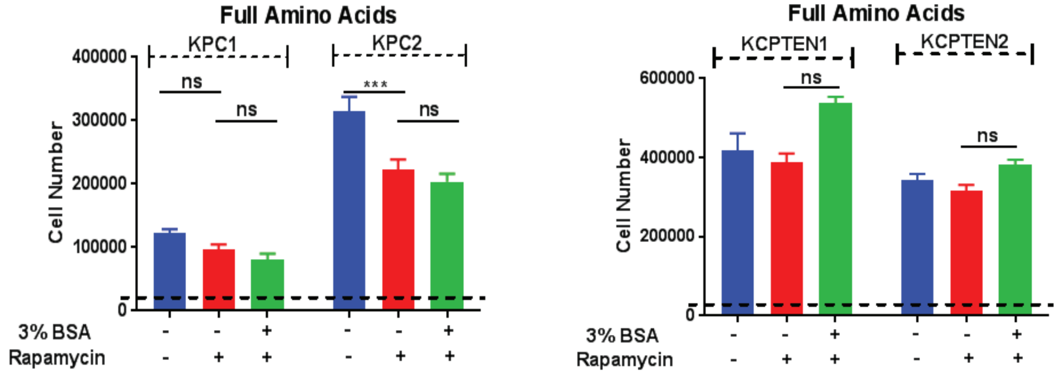

S3.G

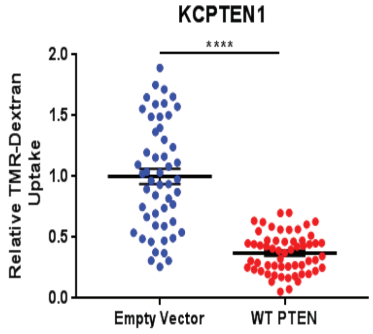

Figure S3. Related to Figure 3.

Protein scavenging confers resistance against mTORC2 inhibition.

- **A** Effect of BSA supplementation on cell proliferation of iBMK-D3 (control) and iBMK-RAS. Cell number was used as a readout for proliferation. Cells were cultured in medium containing 5% EAAs for 72 h.
- **B** Induction of macropinocytosis by RAS activation measured by TMR-Dextran Uptake in iBMK-Ras cells.
- **C** Effect of Torin1 on cell Death was assessed by Annexin V staining. KCPTEN1 and KCPTEN2 cells were cultured in medium containing 5% EAAs ± 3% BSA for 72hours. [Alive: Annexin/PI negative, Apoptotic: Annexin (+)/PI (-) and Necrotic: Annexin V(+)/PI(+)].
- **D** KCPTEN1 and KCPTEN2 cells cultured in 5% EAAs, treated with the dual mTOR inhibitor AZD2014 (1 µM) ± 3% BSA for 72 h. Cell number was used as a readout of proliferation.
- **E** Proliferation assay for KPCs and KCPTENs in 5% EAAs medium treated with 50 nM Rapamycin ± 3% BSA for 72 h.
- **F** Same as **E**, but cells were cultured in full DMEM with standard amino acid concentrations.
- **G** Quantification of TMR-Dextran fluorescence of E. Vector- and WT hPTEN-expressing KCPTEN1 cells. An average of 60 images was acquired per cell line. Values were expressed as relative to the E. Vector control.

For **A** & **B**, error bars represent s.e.m. of 2 biological experiments each conducted with 3 technical replicates. For **D, E & F**, error bars represent s.e.m of 3 biological experiments and significance was determined by two-way ANOVA with Tukey corrections. For **G** error bars represent s.e.m of 3 independent experiments. For TMR-Dextran Assays (**B** & **G**) an average of 50 z-stack images were acquired, and significance was determined by unpaired student's t-test with Welch corrections. ns for non-significant. ****p<0.0001, ***p<0.005, **p<0.05.

Dashed lines indicate the starting cell number.

S4.A

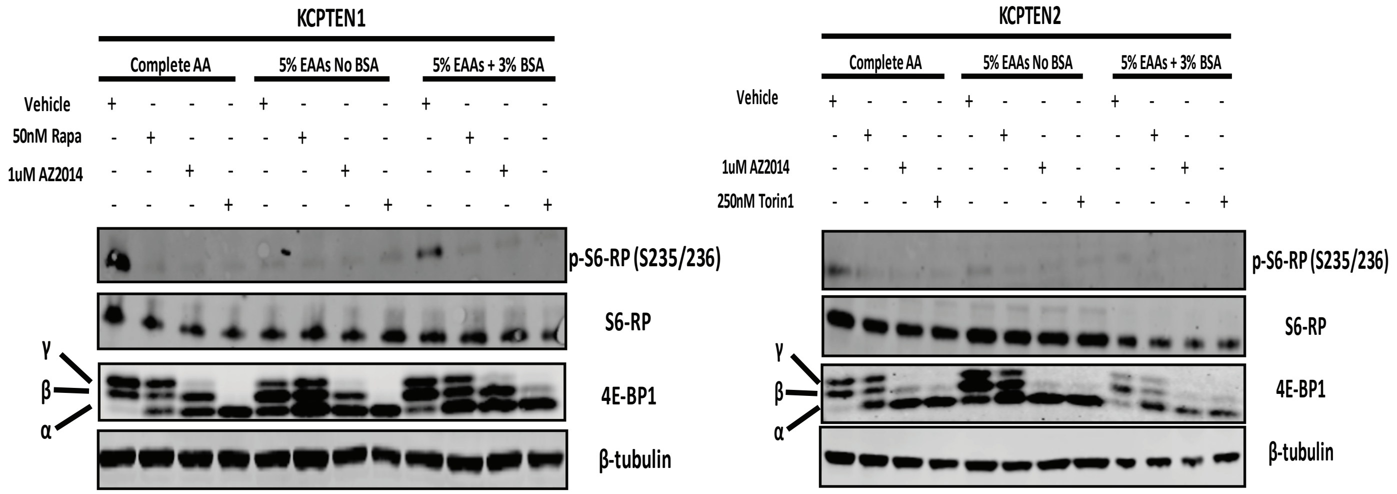

S4.B

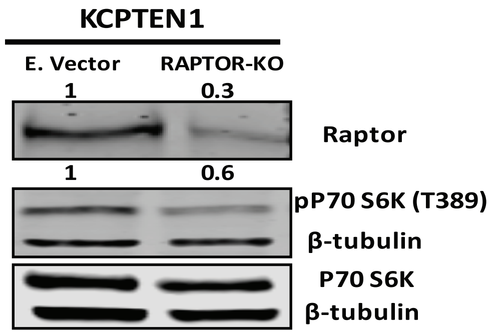

S4.C

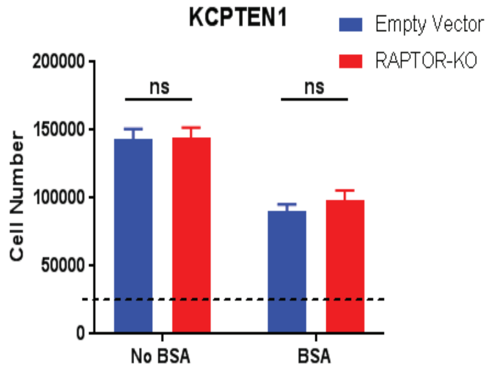

S4.D

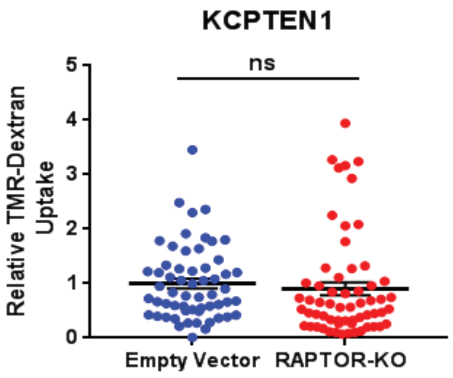

S4.E

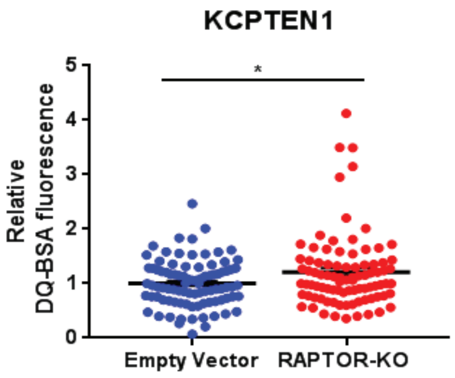

Figure S4. Related to Figure 4.

mTORC1 is not regulating protein scavenging in KCPTEN cells.

- **A** mTORC1 signaling activity upon BSA supplementation and dual mTOR inhibition. Cells were cultured in indicated conditions for 24h and protein levels of mTORC1 downstream targets (pS6-RP, S6-RP and 4E-BP1) were assessed by western blot.
- **B** Raptor, pP70S6K and total P70S6K protein levels in E. Vector- and RAPTOR-KO KCPTEN1 cells. Levels of Raptor were normalized to β-tubulin and values expressed as relative to E. Vector. pP70 S6K was normalized to total P70 S6K and expressed as relative to E. Vector.
- **C** Proliferation assay for RAPTOR-KO KCPTEN1 cells cultured at 5% EAAs ± 3% BSA for 72 hours.
- **D** Quantification of TMR-Dextran uptake and DQ-BSA fluorescence for RAPTOR-KO KCPTEN1 cells. An average of 60 z-stack images was acquired for TMR-Dextran uptake and 90 images for DQ-BSA per cell line. Values were normalized to the average of the Empty Vector-expressing KCPTEN1.

For **C & D** error bars represent s.e.m. of 3 independent experiments. Each biological experiment was performed with 3 technical replicates. Significance was determined by unpaired t-test with Welch's corrections. ns for non-significant, *$p<0.01$

Dashed lines indicate the starting cell number.

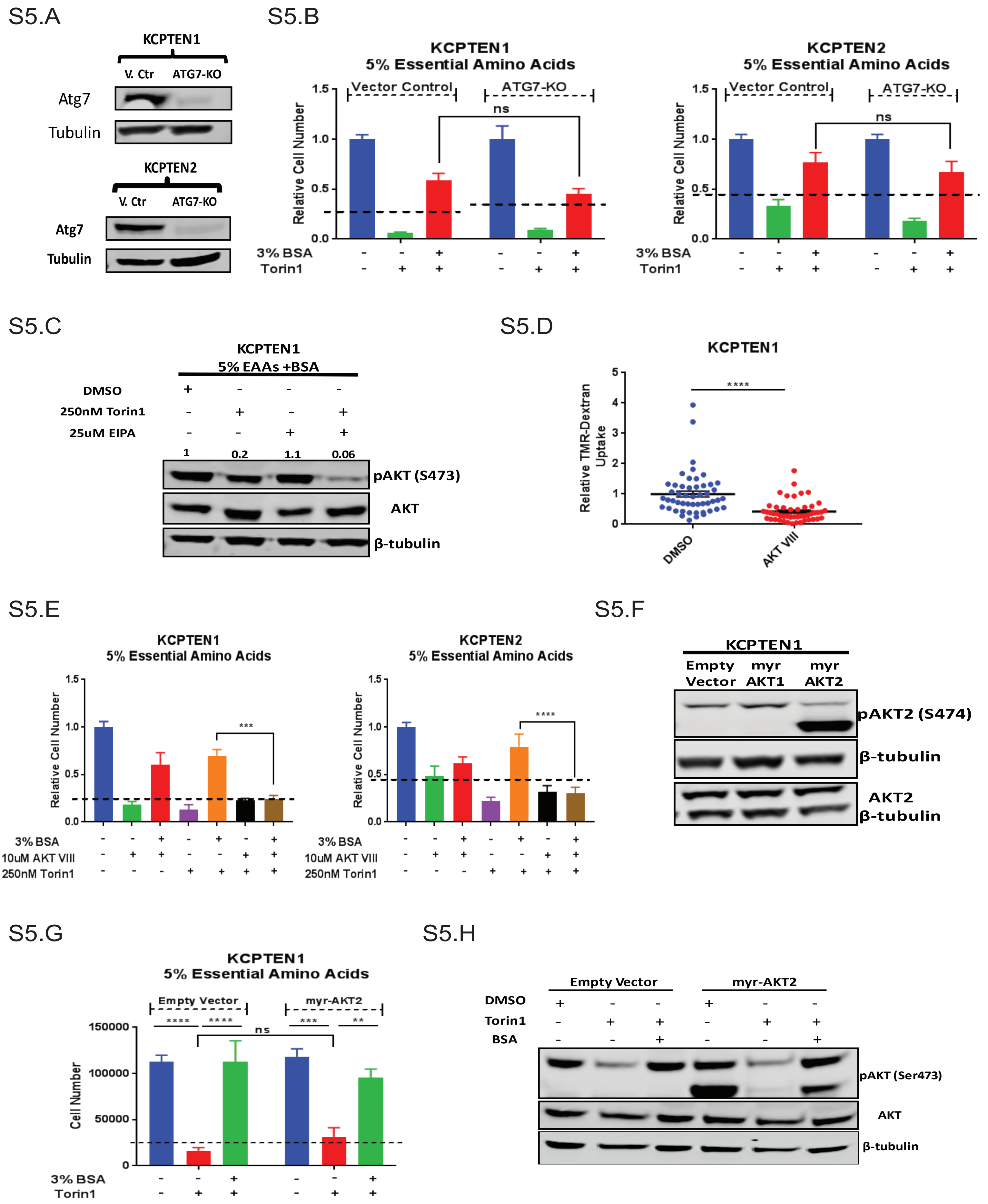
S5.A
KCPTEN1
V. Ctr
ATG7-KO
Atg7
Tubulin
KCPTEN2
V. Ctr
ATG7-KO
Atg7
Tubulin
S5.B
KCPTEN1
5% Essential Amino Acids
Vector Control
ATG7-KO
ns
Relative Cell Number
1.5
1.0
0.5
0.0
3% BSA
Torin1
KCPTEN2
5% Essential Amino Acids
Vector Control
ATG7-KO
ns
Relative Cell Number
3% BSA
Torin1
S5.C
KCPTEN1
5% EAAs +BSA
DMSO
250nM Torin1
25uM EIPA
1
0.2
1.1
0.06
pAKT (S473)
AKT
β-tubulin
S5.D
KCPTEN1
****
Relative TMR-Dextran Uptake
5
4
3
2
1
0
DMSO
AKT VIII
S5.E
KCPTEN1
5% Essential Amino Acids
***
Relative Cell Number
3% BSA
10uM AKT VIII
250nM Torin1
KCPTEN2
5% Essential Amino Acids
****
Relative Cell Number
3% BSA
10uM AKT VIII
250nM Torin1
S5.F
KCPTEN1
Empty Vector
myr AKT1
myr AKT2
pAKT2 (S474)
β-tubulin
AKT2
β-tubulin
S5.G
KCPTEN1
5% Essential Amino Acids
Empty Vector
myr-AKT2
****
****
ns
***
**
Cell Number
150000
100000
50000
0
3% BSA
Torin1
S5.H
Empty Vector
myr-AKT2
DMSO
Torin1
BSA
pAKT (Ser473)
AKT
β-tubulin

Figure S5. Related to Figure 4.

Protein scavenging in PTEN loss-driven PDAC cells is AKT-dependent.

A Western Blot for ATG7 protein levels in KCPTEN1 and KCPTEN2 ATG7-KO cells.

B Proliferation assay for ATG7-KO KCPTEN1 and KC-PTEN2 cells. Cells cultured at 5% of DMEM essential amino acid (EAA) concentrations, treated with 250 nM Torin1 and supplemented with 3% BSA for 72 h.

C pAKT Ser473 and total AKT protein levels in KCPTEN1 cells treated with 25 µM EIPA ± 250 nM Torin1 for 48 hours. pAKT was normalized to total AKT levels and values are expressed relative to DMSO-treated control.

D Quantification of TMR-Dextran uptake for KCPTEN1 cells treated with 20µM of AKT VIII inhibitor for 1 hour. An average of 60 z-stack images was acquired per condition. Values were normalized to the DMSO-treated control.

E Effect of AKT and mTOR inhibition on proliferation of KCPTEN1 and KCPTEN2 cells. Cells were cultured in medium containing 5% EAAs ± 3% BSA and cells treated with 10 µM AKT VIII, 250nM Torin1 and the AKT VIII/Torin1 combination for 72 h.

F pAKT2 (Ser474) and total AKT2 protein levels in Empty Vector- and myrAKT2-expressing KCPTEN1 cells.

G Proliferation assay for KCPTEN1 cells expressing either E. Vector or myrAKT2 cultured in medium containing 5% EAAs ± 3% BSA, treated with 250nM Torin1 for 72 hours.

H pAKT (S473) and total AKT protein levels of KCPTEN1 E. Vector and myrAKT2 cells cultured at the same conditions as (**F**) for 24 hours. The upper band shows phosphorylation of AKT at Serine473 and the lower band indicates the phosphorylated AKT induced by the expression of the myristoylated sequence.

For **B, D & E,** error bars represent s.e.m of 3 biological experiments each conducted with 3 technical replicates. For **B**, significance was determined by unpaired student's t-test with Welch's corrections and for **E & G** significance was determined by Tukey-corrected two-way ANOVA. n-s for non-significant, ****p<0.0001, ***p<0.005, **p<0.05.

S6.A

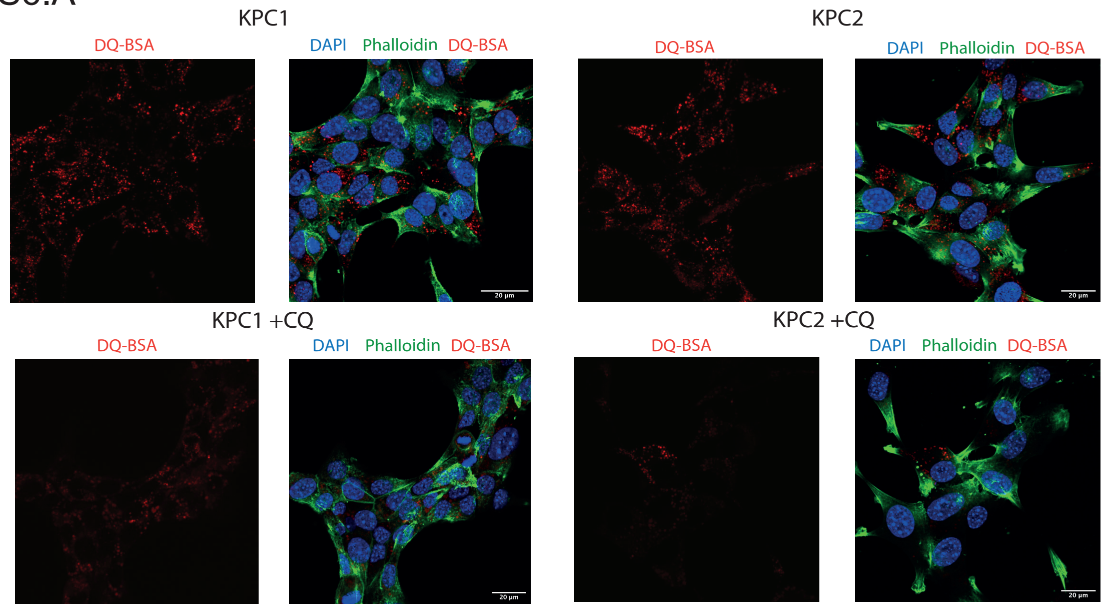

S6.B

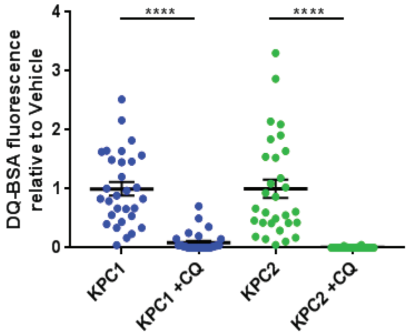

S6.C

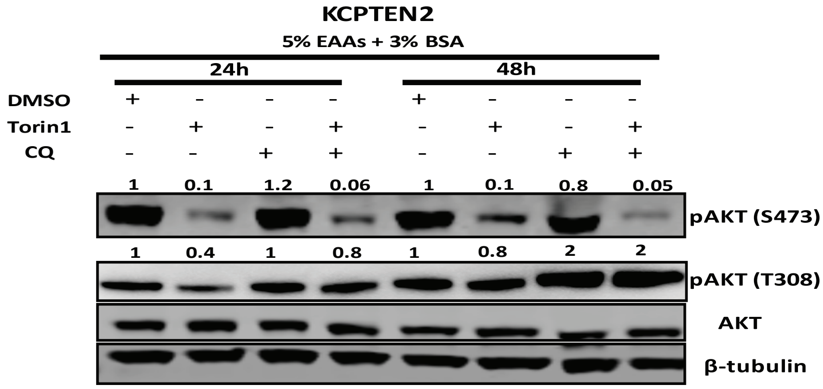

S6.D

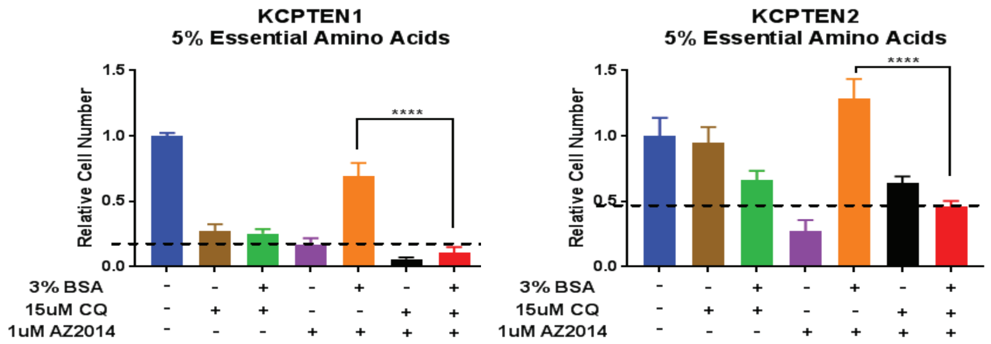

S6.E

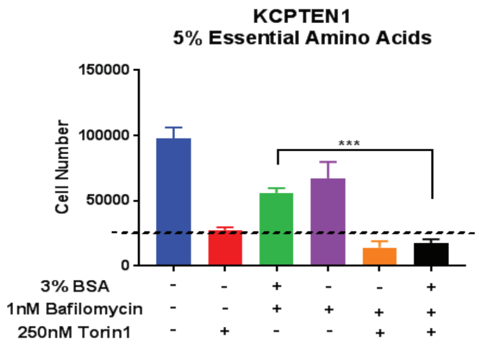

S6.F

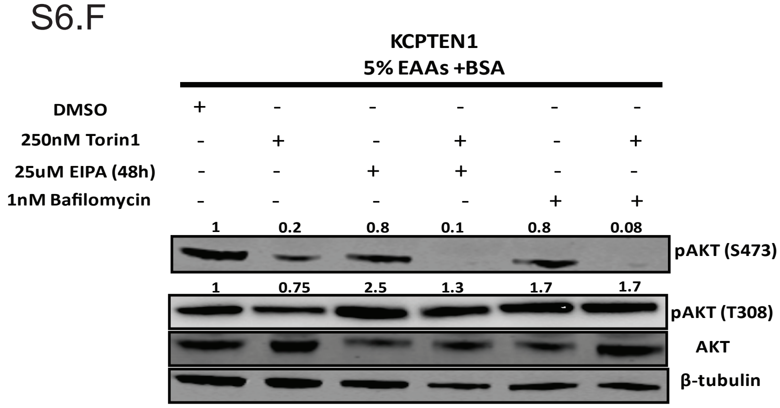

S6.G

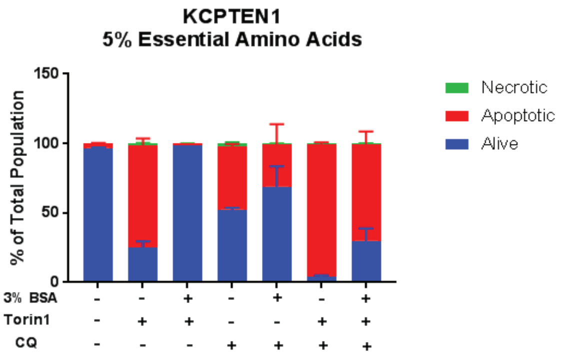

Figure S6. Related to Figure 5. Combined lysosomal and mTOR inhibition alleviates the macropinocytosis-mediated resistance.

- **A** Intracellular degradation of DQ-BSA in KPC1 and KPC2 cells treated with 50 µM Chloroquine (CQ) for 4 h. Alexa 488-Phalloidin was used for F-actin and DAPI for nuclear visualization. Lysosomal inhibition blocks the degradation of extracellular protein.
- **B** Quantification of DQ-BSA fluorescence. An average of 30 z-stack images was acquired per condition. Values were normalized to the DMSO-treated control.
- **C** KCPTEN2 cells were cultured in medium containing 5% EAAs +3% BSA and treated with Torin1, CQ and the combination for 24 and 48. pAKT Ser473 and Thr308 protein levels were assessed by WB. pAKT (S473 and T308) was normalized to the total AKT levels and each value was expressed relative to the vehicle-treated (No BSA) control. Blocking lysosomal activity inhibits recovery of pAKT S473 bypassing the effect of protein supplementation.
- **D** Effect of lysosomal inhibition on proliferation of mTOR-treated KCPTEN1 and KCPTEN2 cells. Cells were cultured in medium containing 5% EAAs ± 3% BSA and cells treated with 15 µM CQ, 1 µM AZD2014 and the CQ/AZD2014 combination for 72 h.
- **E** Same as **D**, but cells were treated with 1 nM Bafilomycin and 250nM Torin1.
- **F** KCPTEN1 cells cultured in medium containing 5% EAAs ± 3% BSA and treated with 25 µM EIPA, 1nM Bafilomycin and the combination for 48 hours. pAKT Ser473 and Thr308 protein levels were assessed by WB. pAKT (S473 & T308) levels were normalized to total AKT and expressed as relative to DMSO-treated control.
- **G** Cell Death was assessed by Annexin V staining. KCPTEN1 cells were cultured in 5% EAAs ± 3% BSA; treated with 250nM Torin1 and 15µM CQ for 72 hours. [Alive: Annexin/PI negative, Apoptotic: Annexin (+)/PI (-) and Necrotic: Annexin V(+)/PI(+)].

For **B**, error bars represent s.e.m for 2 biological experiments each conducted with 3 technical replicates; for **D**, error bars represent s.e.m. of 3 biological experiments; for **G**, a representative experiment is shown, and error bars represent standard deviation (SD). Cell number was normalized to the vehicle-treated (No BSA) control. Scale bar is 10 µm. For **B,** significance was determined by unpaired student's t-test with Welch's corrections and for **D-E,** it was determined by Tukey-corrected two-way ANOVA. ****$p<0.0001$, ***$p<0.005$. Dashed lines indicate the starting cell number.
