# Supplementary material for: Macropinocytosis Renders a Subset of Pancreatic Tumor Cells Resistant to mTOR Inhibition
Source: Cell Rep. 2020 Feb 25;30(8):2729–2742.e4. doi: 10.1016/j.celrep.2020.01.080 (PMC7043007; doi:10.1016/j.celrep.2020.01.080)
Supplement: Document S2. Article plus Supplemental Information [file mmc2.pdf]

Cell Reports

Article

Macropinocytosis Renders a Subset of Pancreatic Tumor Cells Resistant to mTOR Inhibition

Graphical Abstract

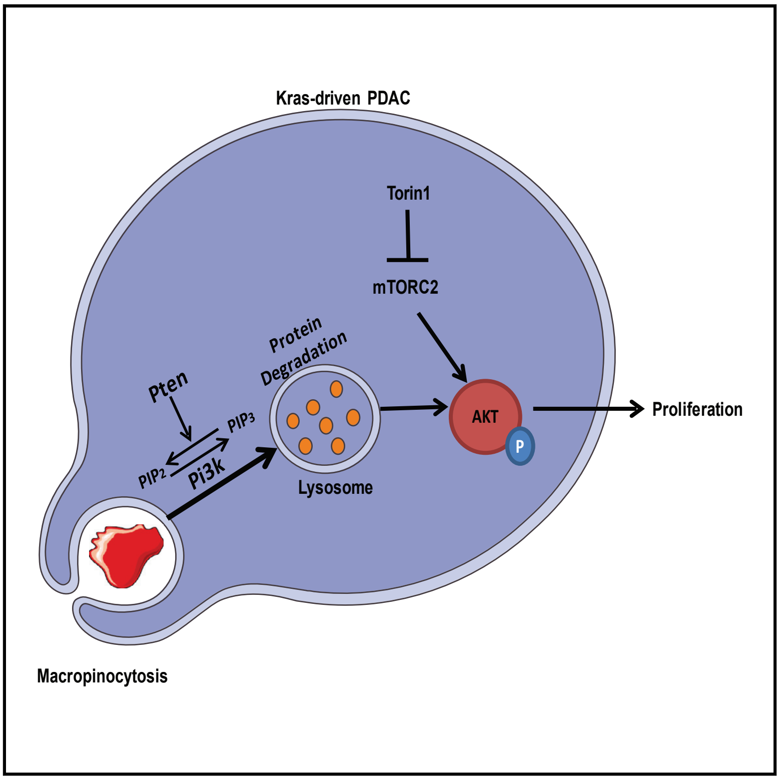

Authors

Evdokia Michalopoulou,
Francesca R. Auciello, Vinay Bulusu, ...,
Jennifer P. Morton, Owen J. Sansom,
Jurre J. Kamphorst

Correspondence

jurre.kamphorst@glasgow.ac.uk

In Brief

Michalopoulou et al. show that in pancreatic cancer cells with oncogenic KRAS and PTEN loss, scavenging of extracellular protein provides resistance against mTOR inhibition. Protein scavenging circumvents mTORC2 signaling by rescuing AKT phosphorylation. Additional treatment with AKT or lysosomal inhibitors strongly potentiates the effect of mTOR inhibitors.

Highlights

- PTEN loss increases scavenging, conferring resistance to mTOR inhibition in PDAC
- mTORC2 selectively regulates macropinocytosis independently of its kinase activity
- Protein scavenging restores pAkt serine 473, leading to recovery of proliferation
- Lysosomal blockade eliminates the protein-mediated resistance to mTOR inhibition

Michalopoulou et al., 2020, Cell Reports *30*, 2729–2742
February 25, 2020 © 2020 The Author(s).
https://doi.org/10.1016/j.celrep.2020.01.080

CellPress

OPEN ACCESS
CellPress

Macropinocytosis Renders a Subset of Pancreatic Tumor Cells Resistant to mTOR Inhibition

Evdokia Michalopoulou,[1,2] Francesca R. Auciello,[1,2] Vinay Bulusu,[1,2] David Strachan,[1] Andrew D. Campbell,[1] Jacqueline Tait-Mulder,[1,2] Saadia A. Karim,[1] Jennifer P. Morton,[1,2] Owen J. Sansom,[1,2] and Jurre J. Kamphorst[1,2,3,4,*]
[1]Cancer Research UK Beatson Institute, Garscube Estate, Switchback Road, Glasgow G61 1BD, UK
[2]Institute of Cancer Sciences, University of Glasgow, Garscube Estate, Switchback Road, Glasgow G61 1QH, UK
[3]Present address: Rheos Medicines, 38 Sidney Street, Cambridge, MA 02139, USA
[4]Lead Contact
*Correspondence: jurre.kamphorst@glasgow.ac.uk
https://doi.org/10.1016/j.celrep.2020.01.080

SUMMARY

Pancreatic ductal adenocarcinoma (PDAC) features a near-universal mutation in *KRAS*. Additionally, the tumor suppressor *PTEN* is lost in ∼10% of patients, and in mouse models, this dramatically accelerates tumor progression. While oncogenic KRAS and phosphatidylinositol 3-kinase (PI3K) cause divergent metabolic phenotypes individually, how they synergize to promote tumor metabolic alterations and dependencies remains unknown. We show that in KRAS-driven murine PDAC cells, loss of *Pten* strongly enhances both mTOR signaling and macropinocytosis. Protein scavenging alleviates sensitivity to mTOR inhibition by rescuing AKT phosphorylation at serine 473 and consequently cell proliferation. Combined inhibition of mTOR and lysosomal processing of internalized protein eliminates the macropinocytosis-mediated resistance. Our results indicate that mTORC2, rather than mTORC1, is an important regulator of protein scavenging and that protein-mediated resistance could explain the lack of effectiveness of mTOR inhibitors in certain genetic backgrounds. Concurrent inhibition of mTOR and protein scavenging might be a valuable therapeutic approach.

INTRODUCTION

Pancreatic ductal adenocarcinoma (PDAC) is a very aggressive type of cancer that, despite its fairly low incidence, is predicted to be the second biggest contributor to cancer deaths by 2030 (Rahib et al., 2014). A near-universal oncogenic driver of PDAC is the constitutive activation of the small GTPase protein KRAS, most often caused by a mutation leading to an inability to hydrolyze guanosine triphosphate (GTP). The resulting constitutive induction of downstream signaling cascades leads to multiple changes that together facilitate rapid cell proliferation, and this includes alterations in cellular metabolism (Bryant et al., 2014). Previous work has shown that oncogenic RAS promotes scavenging of multiple nutrient sources (Guo et al., 2011; Commisso et al., 2013; Kamphorst et al., 2013), which appears to be distinct from oncogenic events leading to constitutive phosphatidylinositol 3-kinase (PI3K)-AKT activation, that promotes *de novo* synthesis of cellular components from glucose and free amino acids, particularly glutamine (Tong et al., 2009). The metabolic scavenging phenotype, induced by KRAS in PDAC, may be especially important for maintaining metabolic plasticity and tumorigenesis in a tumor microenvironment that is poorly vascularized and deprived of primary nutrients like glucose and glutamine (Kamphorst et al., 2015).

One RAS-induced scavenging mechanism that has received considerable attention is macropinocytosis (Commisso et al., 2013). This is an endocytic process that cells use to internalize extracellular material, including protein. After endocytosis, the resulting vesicles, named macropinosomes, which contain the internalized protein, fuse with lysosomes, leading to proteolytic degradation. The freed amino acids generated by this process support the metabolic needs of the cell (Michalopoulou et al., 2016). Scavenging and subsequent hydrolysis of extracellular protein via macropinocytosis was found to support proliferation of KRAS-driven cells in medium devoid of essential amino acids (EAAs) (Kamphorst et al., 2015; Palm et al., 2015). Importantly, macropinocytosis was found to occur both in primary human PDAC specimens (Kamphorst et al., 2015) and in *in vivo* mouse models of PDAC (Davidson et al., 2017).

Although RAS is a main driver of macropinocytosis (Bar-Sagi and Feramisco, 1986), other signaling events are also involved in regulating various aspects of the macropinocytosis cascade. For example, macropinosome formation is dependent on the local production of phosphatidylinositol (3,4,5) triphosphate (PIP3) lipids (Veltman et al., 2016). Consequently, PI3K, which produces PIP3, and its negative regulator, PTEN, were found to regulate lysosomal catabolism of scavenged proteins (Palm et al., 2017). Interestingly, prostate tumor cells, deficient for *PTEN*, survive the nutrient stress by internalizing necrotic bodies through macropinocytosis (Kim et al., 2018). There is also an established connection between PTEN and macropinocytosis through the PI3K target mTORC1. Under nutrient-replete conditions, mTORC1 promotes growth by inducing protein, nucleotide, and fatty acid synthesis while suppressing catabolic processes like autophagy (White and DiPaola, 2009; Howell et al., 2013). In mouse embryonic fibroblasts (MEFs) harboring a

© 2020 The Author(s).
This is an open access article under the CC BY license (http://creativecommons.org/licenses/by/4.0/).

KRAS-activating mutation, it was found that mTORC1 inhibited catabolism of extracellular protein. Consequently, mTORC1 inhibition induced albumin-facilitated proliferation during amino-acid-depleted conditions (Palm et al., 2015). It was later proposed, however, that this did not occur through release of inhibition of lysosomal protein degradation but rather because reduced mTORC1 signaling balances the demand of amino acids required for protein synthesis with their supply via macropinocytosis (Nofal et al., 2017). These recent findings may at least partly explain why mTORC1-directed therapies are ineffective in PDAC (Wolpin et al., 2009; Javle et al., 2010).

Recent research shows that the other arm of the mTOR pathway, mTORC2, is also important in PDAC progression (Driscoll et al., 2016). The mTORC2 complex regulates a wide range of important cellular functions such as cell growth and survival, metabolism, and actin cytoskeleton organization, mainly through AKT phosphorylation at serine 473 that leads to activation of downstream targets such as protein kinase C (PKC) and serum and glucocorticoid-regulated kinase (SGK) (Cybulski and Hall, 2009). AKT can also be phosphorylated at threonine 308 by another kinase, phosphoinositide-dependent kinase 1 (PDK1), which leads to induction of mTORC1 signaling pathway. Full activation of AKT requires phosphorylation of both serine 473 and threonine 308 (Vadlakonda et al., 2013). The induction of different downstream signaling cascades indicates the different cellular functions served by AKT. In human PDAC, AKT phosphorylation at serine 473 has been significantly associated with poor survival (Kennedy et al., 2011). Importantly, a connection between the mTORC2-AKT pathway and macropinocytosis has thus far not been reported.

PTEN deficiency occurs in ~10% of PDAC cases, on top of a near-universal *KRAS* mutation (Ying et al., 2011), and these tumors are highly proliferative (Hill et al., 2010; Kennedy et al., 2011; Rosenfeldt et al., 2017). Here, we investigated how these oncogenic lesions synergized to induce metabolic alterations in PDAC cells using tumor cells derived from the KCPTEN (*Kras* activation and *Pten* loss) genetically engineered mouse model of PDAC (Kennedy et al., 2011; Morran et al., 2014). These cells proliferated more rapidly than cells with wild-type *Pten* and were more sensitive to mTOR inhibition. *Pten* loss also increased protein scavenging, and this was mTORC2 rather than mTORC1 dependent. Surprisingly, albumin supplementation rescued cell proliferation during mTOR inhibition in these cells. Mechanistically, macropinocytosis of albumin recovered AKT phosphorylation at serine 473 and restored growth in an mTORC2 signaling-independent manner. Combining mTOR inhibition with the lysosomal inhibitor chloroquine abrogated the rescue by albumin, leading to extensive cell death. Combinatorial inhibition of mTORC2 and protein scavenging might be a good strategy for treating a subset of PDAC tumors with activated KRAS and PTEN loss.

RESULTS

Pten Loss in KRAS-Driven PDAC Cells Accelerates Proliferation and Causes Dependency on mTOR Signaling

KRAS is nearly always mutated in PDAC, leading to its constitutive activation (Hruban et al., 2000). In addition to *KRAS*, the tumor suppressor *TP53* is mutated in 50%–70% of human PDAC tumors (Scarpa et al., 1993). The effects of these genetic alterations have been modeled in the *Pdx1-Cre; Kras*$^{G12D/+}$; *Trp53*$^{R172H/+}$ (KPC) mouse model (Hingorani et al., 2005), which has been found to recapitulate many of the salient features of human PDAC. More recently, it was found that 10%–15% of PDAC patients display high mTOR phosphorylation (and hence activation) due to either loss of *PTEN* or activating mutations in the *PIK3CA* gene (Schönleben et al., 2006; Ying et al., 2011), and this is associated with extremely poor prognosis (Garcia-Carracedo et al., 2013). Importantly, *Pten* loss came up in two independent studies where transposon-mediated mutagenesis screens were carried out in PDAC mouse models to identify novel partners of oncogenic RAS that accelerate tumor growth (Mann et al., 2012; Pérez-Mancera et al., 2012). Also, *Pdx1-Cre; Kras*$^{G12D/+}$; *Pten*$^{flox/+}$ (KCPTEN) mice exhibit significantly faster tumor progression than KPC mice (Hill et al., 2010; Morran et al., 2014).

The fact that tumor progression is more rapid in KCPTEN mice than KPC mice indicated to us that the combination of mutant *Kras* and *Pten* loss may induce metabolic alterations that facilitate rapid cell growth. In a variety of cell types, loss of the tumor suppressor *PTEN* was found to cause constitutive activation of the downstream PI3K pathway member AKT (Georgescu, 2010). Using KCPTEN cells, we confirmed an increase in phosphorylation of AKT and its immediate downstream target, PRAS40 (Figure 1A). PRAS40 phosphorylation can be mediated by either AKT or the mechanistic target of rapamycin complex 1 (mTORC1), leading to its dissociation from mTORC1, relieving its inhibitory effect (Wiza et al., 2012). Consistent with that, we also found increased levels of further downstream targets, p70S6K/S6-RP, both of which are indicators of increased mTORC1 activity (Figure 1A). Therefore, mTOR is constitutively activated in KCPTEN cells. Furthermore, to determine if increased proliferative capacity is maintained in culture, we compared cell proliferation rates of two KCPTEN cell lines with two KPC cell lines, with each cell line derived from a different mouse (Figure 1B). These results confirmed KCPTEN cell lines proliferate more rapidly than cells with wild-type *Pten*.

mTOR is a key player in a large array of cellular processes, including metabolism, survival, and proliferation, and has been intensely investigated as a therapeutic target. mTOR integrates signaling cues and nutrient availability to regulate growth (Laplante and Sabatini, 2012). It has been found to induce anabolic metabolism, including *de novo* synthesis of nucleotides, proteins, and lipids. Indeed, we found that KCPTEN cells exhibit upregulated fatty acid biosynthesis (Figure S1A). Stemming from this, we investigated whether the KCPTEN cells, due to the constitutive mTOR signaling, would be sensitive to mTOR inhibition in conditions of differential nutrient availability. To test this, we cultured both KPC and KCPTEN cells in standard Dulbecco's modified Eagle's medium (DMEM) containing full amino acids (complete media) and treated the cells with the mTORC1/2 inhibitors Torin1 or AZD2014, using concentrations that led to inhibition of mTOR signaling (Figure S1B). Proliferation was significantly reduced in both cell types, although it was more pronounced in KCPTEN cells (Figure 1C). We next assessed the effect of mTOR inhibition in medium containing 5% of the

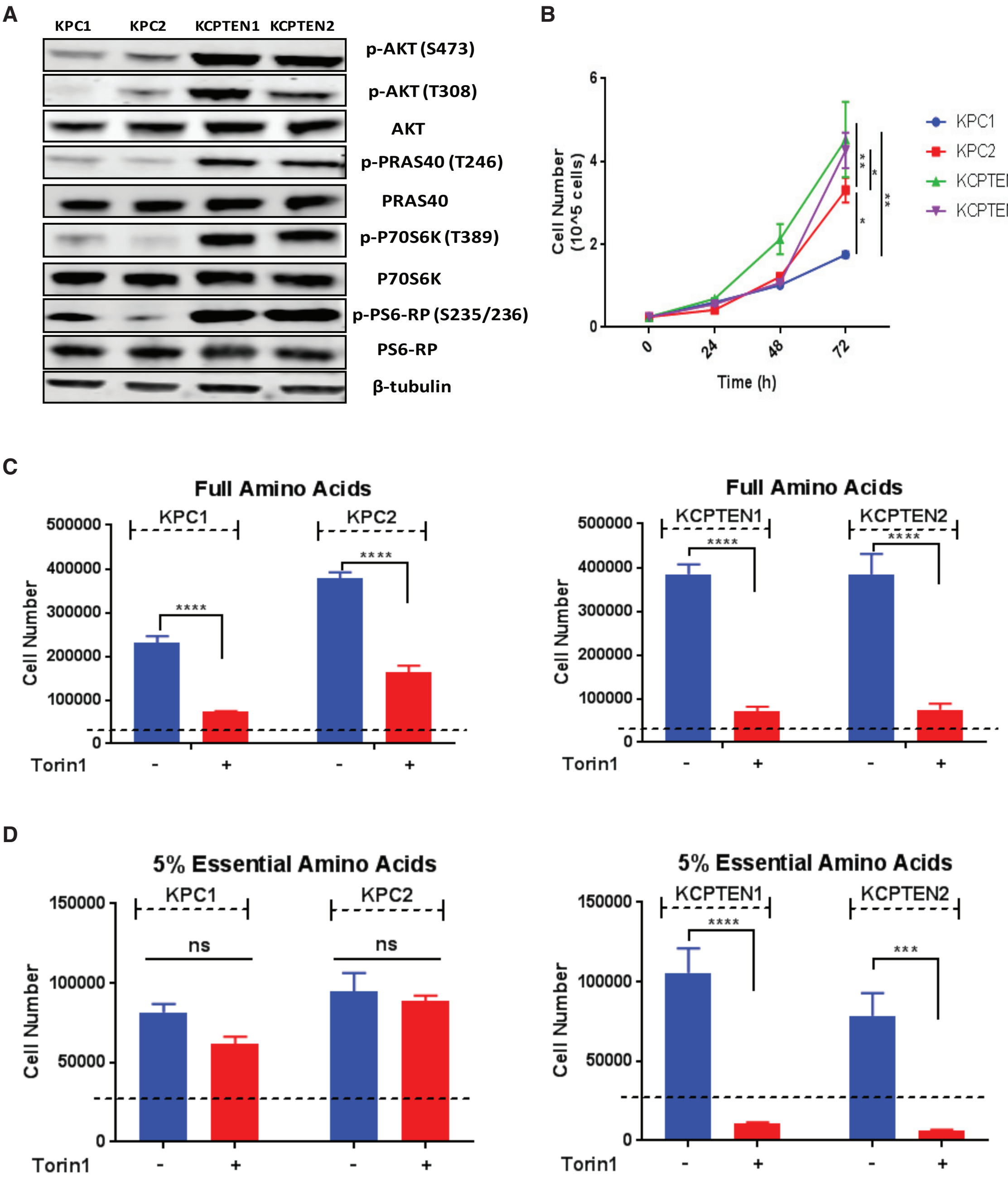

Figure 1. KCPTEN Cells Are Exquisitely Sensitive to mTOR Inhibition

(A) Comparison of mTORC1 and AKT signaling in KPC and KCPTEN cells.

(B) Time-course proliferation assay of KPC and KCPTEN cells.

(C) Effect of dual mTOR inhibition (250 nM Torin1) on proliferation of KPC and KCPTEN cells. Cells were cultured in full DMEM with standard amino acid concentrations and treated with Torin1 for 72 h.

(D) As for (C), but with 5% of the standard DMEM essential amino acid (EAA) concentrations.

For (B)–(D), error bars represent SEM of three biological experiments, each conducted with three technical replicates. Significance was determined by one-way ANOVA (B) or two-way ANOVA with Tukey corrections (C and D). ns, non-significant. ****p < 0.0001, ***p < 0.001, **p < 0.05, and *p < 0.01. Dashed lines indicate the starting cell number.

original EAA levels, including the well-known mTOR regulator leucine (Lynch et al., 2000). While proliferation of KPC cells was lower in these conditions than in complete medium, it was not further affected by mTOR inhibition. In contrast, the KCPTEN cells remained exquisitely sensitive to mTOR inhibition despite the low EAA levels (Figures 1D and S1C). Thus, *Pten* deficiency,

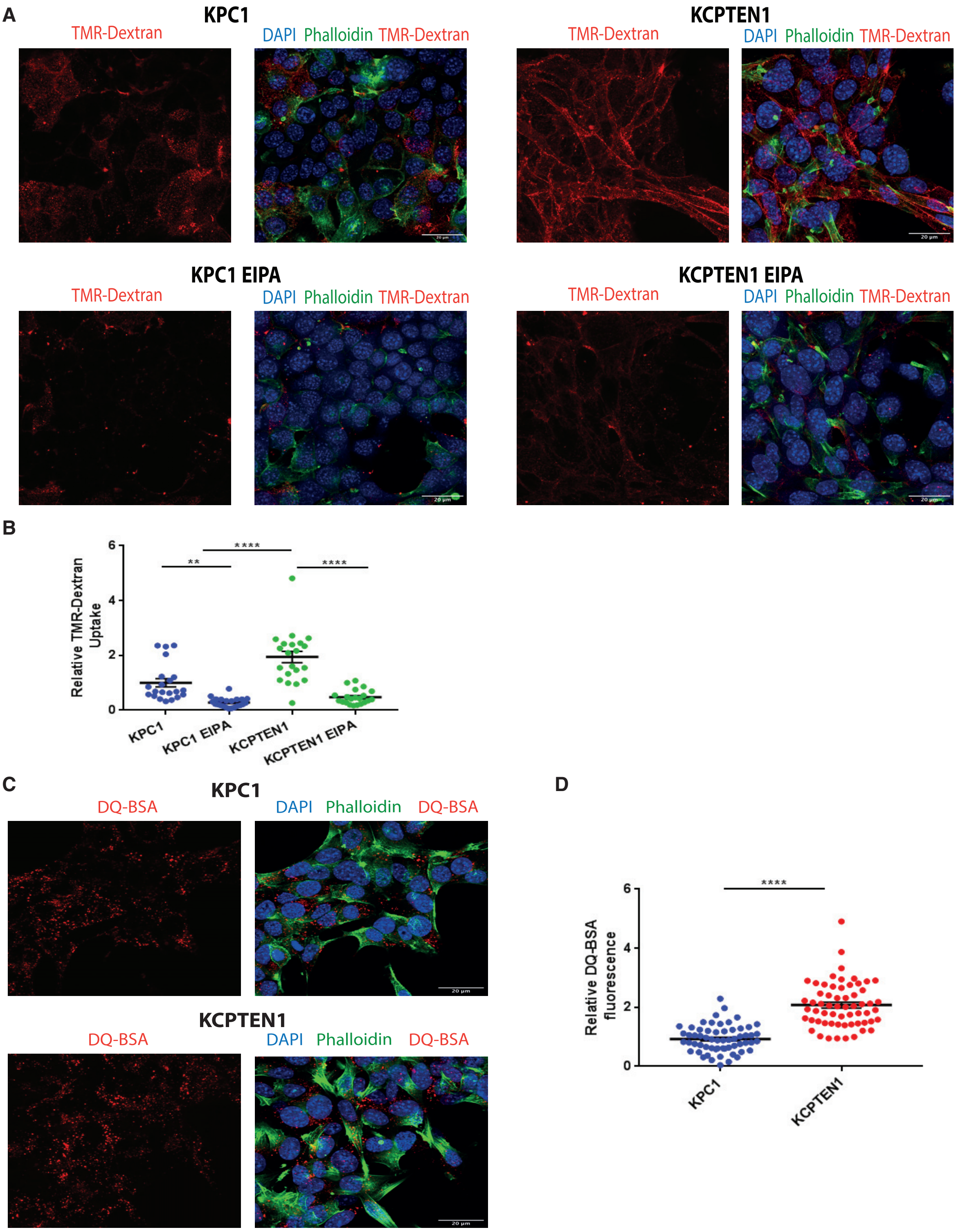

Figure 2. PTEN Loss in KRAS-Driven PDAC Tumor Cells Upregulates Macropinocytosis

(A) Macropinocytosis assay using TMR-dextran as a marker of macropinosomes (red staining) in KPC and KCPTEN cells. Nuclei stained with DAPI (blue) (4′,6-diamidino-2-phenylindole) and Alexa 488 phalloidin (green) for actin staining (cellular periphery). The amiloride macropinocytosis inhibitor EIPA was used as a control at 50 μM.

(B) Quantification of TMR-dextran fluorescence. An average of 30 z stack images was acquired per condition. Values were normalized to the average of the vehicle-treated (DMSO) control.

(legend continued on next page)

which is also known to activate several downstream pathways, sensitizes to mTOR inhibition, regardless of free amino acid availability.

Pten Loss in KRAS-Driven PDAC Tumor Cells Enhances Macropinocytosis

Cells are able to take up amino acids in the free, monomeric, form. Additionally, PDAC cells are able to acquire amino acids through scavenging and subsequent hydrolysis of extracellular protein (Commisso et al., 2013; Kamphorst et al., 2015). This occurs via a process called macropinocytosis, which is induced by oncogenic RAS (Commisso et al., 2013; Kamphorst et al., 2015). The process of macropinocytosis has been studied in depth in the model organism *Dictyostelium discoideum* (Dumontier et al., 2000; Bloomfield et al., 2015), and multiple studies clearly defined a role for PI3K and its product, PIP3, in macropinosome formation (Veltman et al., 2016). Only recently, studies emerged providing an initial link between the tumor suppressor PTEN and protein scavenging in MEFs and prostate cancer cells (Palm et al., 2017; Kim et al., 2018). Based on these observations, we interrogated whether *Pten* loss, concurrent to causing constitutive activation of anabolic mTOR signaling, could lead to increased macropinocytosis in PDAC cells. We therefore used the high-molecular-weight polysaccharide tetramethylrhodamine (TMR)-dextran, which represents an established marker for active macropinocytosis (Commisso et al., 2014), to assess the ability of both KPC and KCPTEN cells to take up extracellular material. Although KPC cells displayed active macropinocytosis, we observed that both KCPTEN cell lines had significantly higher macropinocytic activity than KPC cells (Figures 2A, 2B, S2A, and S2B), confirming the role of PTEN and consequently PI3K in macropinocytosis. As expected, dextran uptake through macropinocytosis was significantly inhibited in both KPC and KCPTEN in the presence of the reportedly selective inhibitor of macropinocytosis, ethylisopropyl amiloride (EIPA) (Figures 2A, 2B, S2A, and S2B). We validated this further using cytochalasin D, which is also known to block macropinocytosis (Mercer and Helenius, 2009; Recouvreux and Commisso, 2017) (Figure S2C). Following ingestion, extracellular protein is hydrolyzed in the lysosomes, generating free amino acids, which then feed into cellular metabolic pathways. Thus, we tested if lysosomal protein hydrolysis is also induced upon *Pten* loss. For this, we used fluorogenic dequenched bovine serum albumin (DQ-BSA), which self-quenches when intact but starts to fluoresce upon lysosomal digestion (Commisso et al., 2013). Using this assay on KPC and KCPTEN cells (Frost et al., 2017), we observed that fluorescence was strongly increased in KCPTEN cells (Figures 2C, 2D, S2D, and S2E). Thus, in the setting of *Kras*-driven PDAC cells, PTEN deficiency increases protein scavenging and hydrolysis.

Protein Scavenging Is Regulated by mTORC2

Our results so far show that *Pten* deficiency leads to both increased anabolic mTOR signaling and protein scavenging through macropinocytosis. Additionally, work by others showed a direct link between mTOR and macropinocytosis (Palm et al., 2015; Nofal et al., 2017). For these reasons, we next sought to investigate the sensitivity of our PDAC cells to mTOR inhibition in the absence or presence of extracellular bovine serum albumin (BSA). While KPC cells proliferate in low-EAA conditions, mTOR inhibition had no further effect, nor did BSA supplementation (Figure 3A). This is different from previous work where it was shown that *Ras*-driven MEFs do not proliferate under conditions of deprived EAAs, a phenotype that can be rescued in the presence of extracellular protein (Palm et al., 2015; Nofal et al., 2017). To determine if the observed difference between the two systems could be explained by the genetic diversity across the different cell types, we cultured immortalized baby mouse kidney cells (iBMK) harboring a *Ras*-activating mutation (Degenhardt et al., 2002; Degenhardt and White, 2006) and similarly found they were still proliferating upon albumin supplementation (Figure S3A). Increased TMR-dextran uptake was observed in iBMK-RAS cells, confirming that oncogenic RAS induces macropinocytosis (Figure S3B). Stemming from the above, we conclude that the genetic background of cell lines can explain differences in both resistance to amino acid deprivation and the ability to proliferate with exogenous protein as an amino acid source in certain conditions.

Next, we performed a head-to-head comparison of the ability of KPC and KCPTEN cells to proliferate using protein in both low-EAA and replete-medium conditions, in the presence or absence of mTOR inhibition. We used cell number as a readout of proliferation. Surprisingly, while mTOR inhibition and protein supplementation had no effect on KPC cells in low EAA, in KCPTEN cells, mTOR inhibition caused a growth defect, and this was rescued by protein supplementation (Figure 3A). Furthermore, we found that mTOR inhibition induced cell death via apoptosis, as assessed by Annexin V staining, which was bypassed upon albumin supplementation (Figure S3C). In replete-medium conditions with full EAA, proliferation of KPC cells was significantly affected by mTOR inhibition, but albumin did not rescue this (Figure 3B). In KCPTEN cells, however, the presence of albumin again substantially rescued proliferation (Figures 3B and S3D). Overall, this argues that macropinocytosis is a mechanism by which KCPTEN cells overcome metabolic crisis and become resistant to mTOR inhibition, independently of free amino acid availability.

To investigate more in depth the role of *Pten* deficiency in contributing to the obtained resistance against mTOR inhibitors, we expressed human PTEN in KCPTEN1 cells (Figure 3C). We found that KCPTEN cells expressing hPTEN were still sensitive to mTORC1/2 inhibition, and protein supplementation could still rescue the observed proliferation defects. However, the recovery of proliferation in the presence of albumin was significantly decreased in hPTEN-expressing cells compared to the empty vector KCPTENs, indicating that *Pten* expression partially blocks the proliferative advantage conferred by protein scavenging

(C) Lysosomal processing of extracellular protein uptake via macropinocytosis was assessed by DQ-BSA fluorescence.
(D) Quantification of DQ-BSA fluorescence. An average of 50 z stack images were acquired per cell line. Values were normalized to the average value of KPC1. For (B) and (D), error bar represents SEM of two biological experiments, each conducted with three technical replicates. Scale bar represents 20 μm. Significance was determined by one-way ANOVA. ns, non-significant. ****$p < 0.0001$ and ***$p < 0.001$.

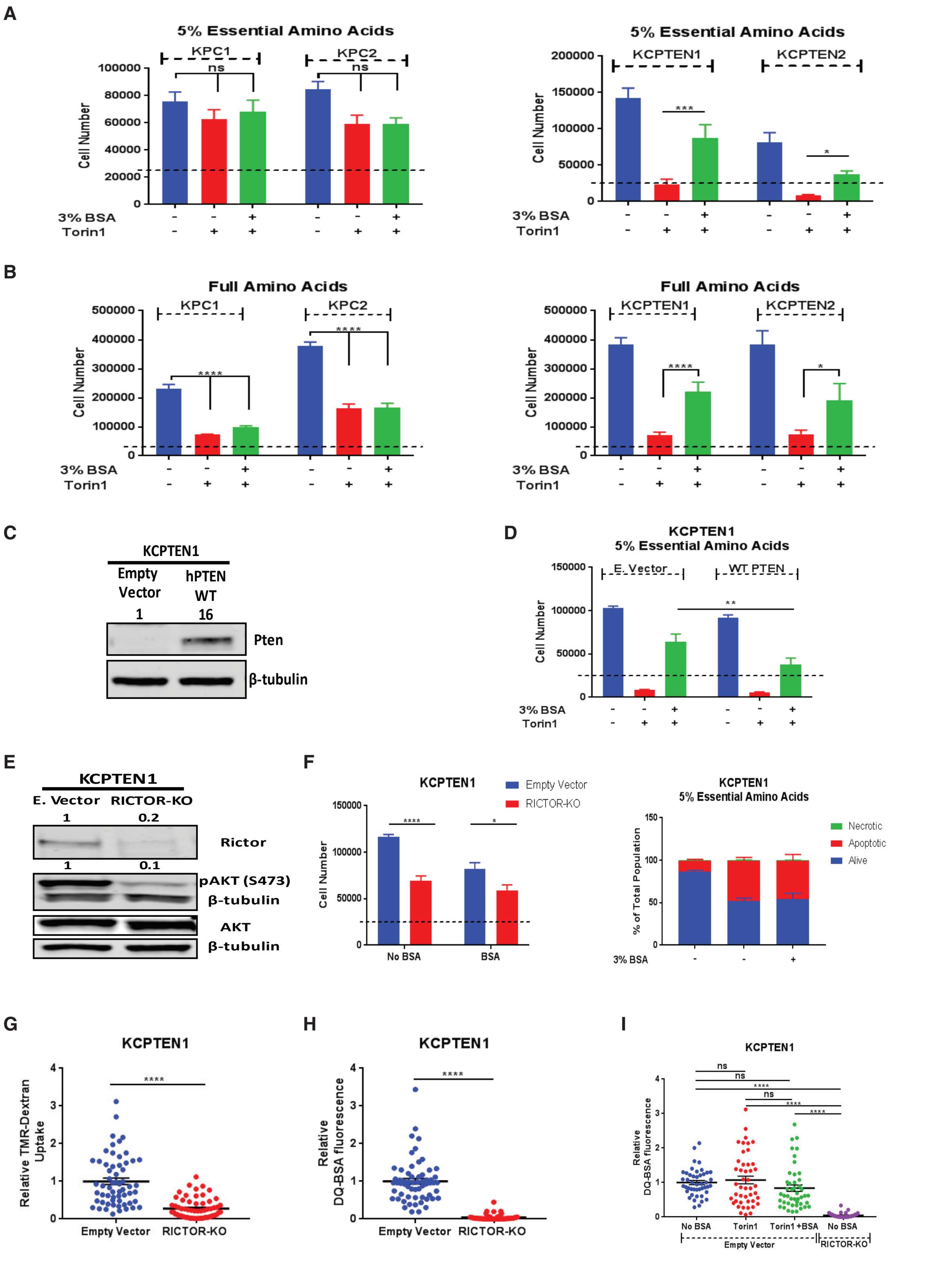

(legend on next page)

(Figure 3D). Indeed, PTEN expression significantly decreased the uptake of extracellular material via macropinocytosis as assessed by TMR-dextran (Figure S3G). Overall, our data suggest that on top of the oncogenic KRas activation, *Pten* deficiency increases macropinocytosis while its reexpression partially suppresses the ability to use extracellular protein to proliferate.

mTORC1 has been argued to regulate hydrolysis of scavenged protein (Palm et al., 2015), and more recently, it was shown that (partial) mTORC1 inhibition balances amino acid demand for growth with amino acid availability via macropinocytosis (Palm et al., 2015; Nofal et al., 2017). Importantly, mTORC2 is also known to play a significant role in PDAC (Driscoll et al., 2016), but its exact role remains understudied. The mTOR inhibitors we used thus far (torin1 and AZD2014) are ATP-competitive inhibitors of both mTORC1 and mTORC2. We therefore asked if the induced cell death in KCPTEN cells was mTORC1 specific. Rapamycin specifically blocks mTORC1 activity, but surprisingly, there was no effect on proliferation of KCPTEN cells, either in complete medium or in medium with low EAAs (Figures S3E and S3F). This suggested that KCPTEN cells are dependent on mTORC2 rather than mTORC1 signaling and that this dependency can be overcome by macropinocytosis. To further investigate the role of mTORC1, we asked if protein supplementation was able to re-engage mTORC1 downstream targets (Figure S4A). Both Torin1 and AZD2014 were capable of blocking mTOR activity, as evidenced by an abrogation of phosphorylation of its downstream target, S6-RP. Furthermore, phosphorylation of 4E-BP1, as indicated by the hyperphosphorylated isoforms β and γ, was also blocked by the mTOR-dual inhibitors (Figure S4A). Protein supplementation did not rescue the phosphorylation of any of the mTORC1 targets, providing further credence to the notion that the rescue of impaired proliferation occurs independently of mTORC1. Furthermore, using CRISPR/Cas9 technology, we created mTORC1-deficient KCPTEN cells by ablating RAPTOR, which is an essential subunit of the complex (Figure S4B). Confirming our findings with mTORC1 inhibitors (rapamycin), RAPTOR-knockout KCPTEN cells displayed no significant differences in proliferation under low-EAA conditions (Figure S4C) or macropinocytosis levels (Figure S4D) compared to RAPTOR wildtype cells. However, we observed increased lysosomal processing measured by DQ-BSA fluorescence (Figure S4E), which has also been reported by studies in RAPTOR-knockout MEFs (Palm et al., 2015). Together, these findings indicate that the observed proliferation mediated by protein scavenging is mTORC1 independent.

Our results indicate that KCPTEN cells display sensitivity to mTORC2 inhibition. To evaluate the role of mTORC2 we created mTORC2-deficient KCPTEN cells by silencing RICTOR, an essential subunit of mTORC2 (Figure 3E). We found that KCPTEN RICTOR-knockout cells were somewhat more sensitive to the low-EAA environment, and protein supplementation could not rescue the observed proliferation defects (Figure 3F). RICTOR deficiency also led to induction of apoptosis, which could not be alleviated by protein supplementation either (Figure 3F). This aligned with our observation that uptake of TMR-dextran via macropinocytosis was significantly decreased (Figure 3G) and DQ-BSA was completely abolished in RICTOR-knockout KCPTENs compared to the empty vector (Figure 3H). These results implicate a dominant role of mTORC2 in mediating protein scavenging.

Our experiments with inhibitors and genetic knockout both demonstrate the involvement of mTORC2 in protein scavenging. They also highlight a difference, with pharmacological inhibition of mTORC2 (Torin1) leading to defects in proliferation that can be rescued by protein scavenging and CRISPR/Cas9-mediated knockout blocking protein scavenging altogether. Genetic ablation of RICTOR, in addition to blocking the kinase activity of mTORC2 like the inhibitors, also disrupts the entire mTORC2 scaffold. To investigate more in depth the effect of the two modes of mTORC2 disruption on protein scavenging, we utilized the DQ-BSA assay and compared Torin1-treated KCPTEN cells with RICTOR-knockout KCPTEN cells. Torin1 treatment did not impair protein scavenging (DQ-BSA fluorescence), regardless of protein supplementation. In RICTOR-deficient KCPTEN cells, however, protein scavenging was abolished (Figure 3I). Overall, these data clearly demonstrate an intricate interplay between mTORC2 and protein scavenging that extends beyond its function as a kinase. In this regard, it is worth noting that mTORC2

Figure 3. Protein Scavenging Is Regulated by mTORC2

(A) Effect of bovine serum albumin (BSA) supplementation (3%) on cell proliferation during Torin1 inhibition. Assay was done in media containing 5% EAAs for 72 h (250 nM Torin1).

(B) As (A), but with standard DMEM amino acid concentrations.

(C) Pten protein levels in empty-vector- and hPTEN-expressing KCPTEN1 cells.

(D) Same as (A), but for hPTEN-expressing KCPTEN1 cells.

(E) RICTOR, pAKT serine 473, and total AKT protein levels in empty-vector- and RICTOR-knockout (RICTOR-KO) KCPTEN1 cells. RICTOR levels were normalized to β-tubulin and expressed relative to the empty vector. pAKT (serine 473) was normalized to total AKT levels and expressed relative to the empty vector.

(F) Cell number and apoptosis as readouts of proliferation and cell death, respectively, in RICTOR-KO KCPTEN1 cells cultured in 5% EAAs ±3% BSA for 72 h.

(G) Quantification of TMR-dextran uptake and DQ-BSA fluorescence for RICTOR-KO KCPTEN1 cells. An average of 60 z stack images were acquired per condition. Values were normalized to the empty-vector-expressing KCPTEN1.

(H) Same as (G), but for DQ-BSA fluorescence.

(I) Quantification of DQ-BSA fluorescence in KCPTEN1 E empty vector and RICTOR-KO cells. Empty vector cells were treated with Torin1 ±3% BSA for 24 ho. An average of 45 z stack images were acquired per condition. Values were normalized to the untreated (no BSA) empty-vector-expressing KCPTEN1.

For (A), (B), (D), (G), and (H), error bars represent SEM of three independent experiments, each with three technical replicates. Significance was determined by Tukey-corrected two-way ANOVA. For (F) (cell number), error bars represent SEM of four biological experiments, each conducted with three technical replicates. For (F) (apoptosis), error bars represent SEM of two biological experiments, each with three technical replicates. For (G) and (H), significance was determined by Student's t test with Welch's corrections. For (I), error bars represent SEM of three biological experiments, each with three technical replicates, and significance was determined by one-way ANOVA with Tukey corrections. ns, nonsignificant. ****$p < 0.0001$, ***$p < 0.0005$ **$p < 0.005$, and *$p < 0.01$. Dashed lines indicate the starting cell number.

has been shown to reside on endosomal vesicles (Ebner et al., 2017).

Protein Scavenging Supports Proliferation through Recovery of AKT Serine 473 Phosphorylation

As protein consumption supports proliferation of KCPTEN cells despite inhibition of mTOR, we were interested in dissecting the underlined molecular mechanisms of rescue of proliferation by protein scavenging in these conditions. Autophagy is a well-described catabolic mechanism often found upregulated in RAS-driven tumors like PDAC. mTOR is a negative regulator of autophagy (Rabinowitz and White, 2010). We therefore asked whether autophagy induction contributes to the recovery of proliferation in KCPTEN cells during mTOR inhibition. For this reason, we utilized the CRISPR/Cas9 technology to create autophagy-deficient KCPTEN cells by knocking out *Atg7*, which is an essential component of autophagosomal membrane formation (Figure S5A). We cultured the KCPTEN ATG7-knockout cells in medium containing 5% EAAs and treated them with Torin1, but proliferation was not affected in any of the two cell lines, suggesting that autophagy does not contribute to the recovery of proliferation (Figure S5B).

We next expanded our analysis of mTORC2-downstream signaling mediators that could potentially explain the recovery of proliferation. We noticed that while phosphorylation in serine 473 of the well-described mTORC2 target AKT was significantly reduced with the mTORC1/2 inhibitors AZD2014 or Torin1, it was restored upon protein supplementation regardless of free amino acid availability (Figures 4A and 4B). We also investigated the effect on the other AKT phosphorylation site, threonine 308, which was not greatly affected compared to the serine 473 site (Figures 4A and 4B). To determine whether the recovery of AKT serine 473 phosphorylation depends on macropinocytosis, we used EIPA in combination with Torin1. Indeed, AKT phosphorylation was significantly decreased upon inhibition of macropinocytosis, demonstrating that AKT phosphorylation is macropinocytosis dependent (Figure S5C). Furthermore, we showed that blocking AKT significantly decreases TMR-dextran uptake via macropinocytosis in KCPTEN cells (Figure S5D). While AKT is well known to induce mTORC1 signaling and can promote cell proliferation in this manner, it could also support proliferation via other signaling cascades (Manning and Toker, 2017). To test if AKT phosphorylation and hence activity upon protein supplementation is at least partially responsible for the rescue of proliferation, we incubated the cells with either of two AKT inhibitors, AZD5363 and AKT VIII, in addition to Torin1 and BSA (Figures 4C and S5E). Interestingly, when used in isolation, AZ5363, which is an ATP-competitive inhibitor, had no effect on proliferation, while AKT VIII, an allosteric inhibitor, reduced proliferation, indicating a mechanistic difference in the displayed suppression profiles of the inhibitors. However, in both cases, BSA-mediated restoration of cell proliferation of mTOR-inhibited cells was greatly diminished with AKT inhibition, consistent with the notion that restoration of AKT phosphorylation by protein supplementation is an important contributor to the observed rescue of cell proliferation. Thus, protein scavenging restores growth in mTOR-inhibited cells by reengaging AKT phosphorylation and activity.

To further investigate the role of AKT, we expressed myristoylated AKT2 (myrAKT2) in KCPTEN1 cells (Figure S5F). myrAKT2-expressing cells lack the pleckstrin homology (PH) domain where PIP lipids bind, and it can therefore get phosphorylated in a PIP-independent manner (Manning and Toker, 2017). However, myrAKT2 can still be phosphorylated by mTORC2 at its hydrophobic motif, which is intact. To check whether active AKT could confer resistance to mTOR inhibitors, we treated KCPTEN empty-vector- and myrAKT2-expressing cells with Torin1 and supplemented with 3% albumin. We found that KCPTEN1 myrAKT2 cells were still sensitive to mTOR inhibition (Figure S5G). To further investigate this, we checked the effect of mTOR dual inhibition and protein supplementation on AKT phosphorylation levels. In myrAKT2-expressing KCPTEN cells, Torin1 still blocked AKT phosphorylation at serine 473, which was then recovered by protein supplementation similar to the empty-vector-expressing KCPTEN cells (Figure S5H). Thus, myrAKT2 is subject to the same phosphorylation events at the hydrophobic domain, and hence activity regulation, as endogenous AKT upon mTOR inhibition and protein supplementation, lending further credence to our observations.

Combined mTOR and Lysosomal Inhibition Induce Cell Death in KCPTEN Cells

Serine 473 of AKT is a known downstream phosphorylation target of mTORC2 (Sarbassov et al., 2005). Our work shows that this phosphorylation site of AKT is rescued by protein supplementation during mTOR inhibition. The fact that mTORC2 activity is also inhibited with the dual mTOR inhibitors suggests that this phosphorylation event occurs through an alternative mechanism. Scavenging and recycling pathways that converge on the lysosomal pathway have gathered a lot of attention and are considered a hallmark of pancreatic cancer (Perera et al., 2015; Pavlova and Thompson, 2016). Macropinosomes fuse with lysosomes, and the extracellular protein they contain is hydrolyzed to yield free amino acids that feed into metabolic pathways (Michalopoulou et al., 2016). As protein supplementation restores AKT phosphorylation in mTOR-inhibited cells, we wondered if lysosomal processing of extracellular protein is a critical event for AKT phosphorylation. We first confirmed that the proteolytic degradation of extracellular protein is dependent on lysosomes by using chloroquine, a lysosomal inhibitor. This indeed abolished the lysosomal processing, as it is depicted by the loss of DQ-BSA fluorescence in both KPC and KCPTEN cell lines (Figures 5A, 5B, S6A, and S6B).

We next treated KCPTEN cells with both chloroquine and a dual mTOR inhibitor. In the absence of the mTORC1/2 inhibitor Torin1, chloroquine treatment did not affect AKT phosphorylation at serine 473 (Figures 5C and S6C). However, in cells exposed to supplemented protein, persistent AKT phosphorylation upon mTOR inhibition was further abrogated with chloroquine treatment (Figures 5C and S6C). In line with earlier obtained results (Figure 4C), the loss of AKT phosphorylation, this time due to chloroquine treatment, led to an inability of supplemented protein to rescue mTOR-mediated inhibition of proliferation (Figures 5D and S6D). We further validated these results by using another lysosomal inhibitor, bafilomycin, that also abrogated proliferation and AKT phosphorylation upon combination with mTOR inhibition

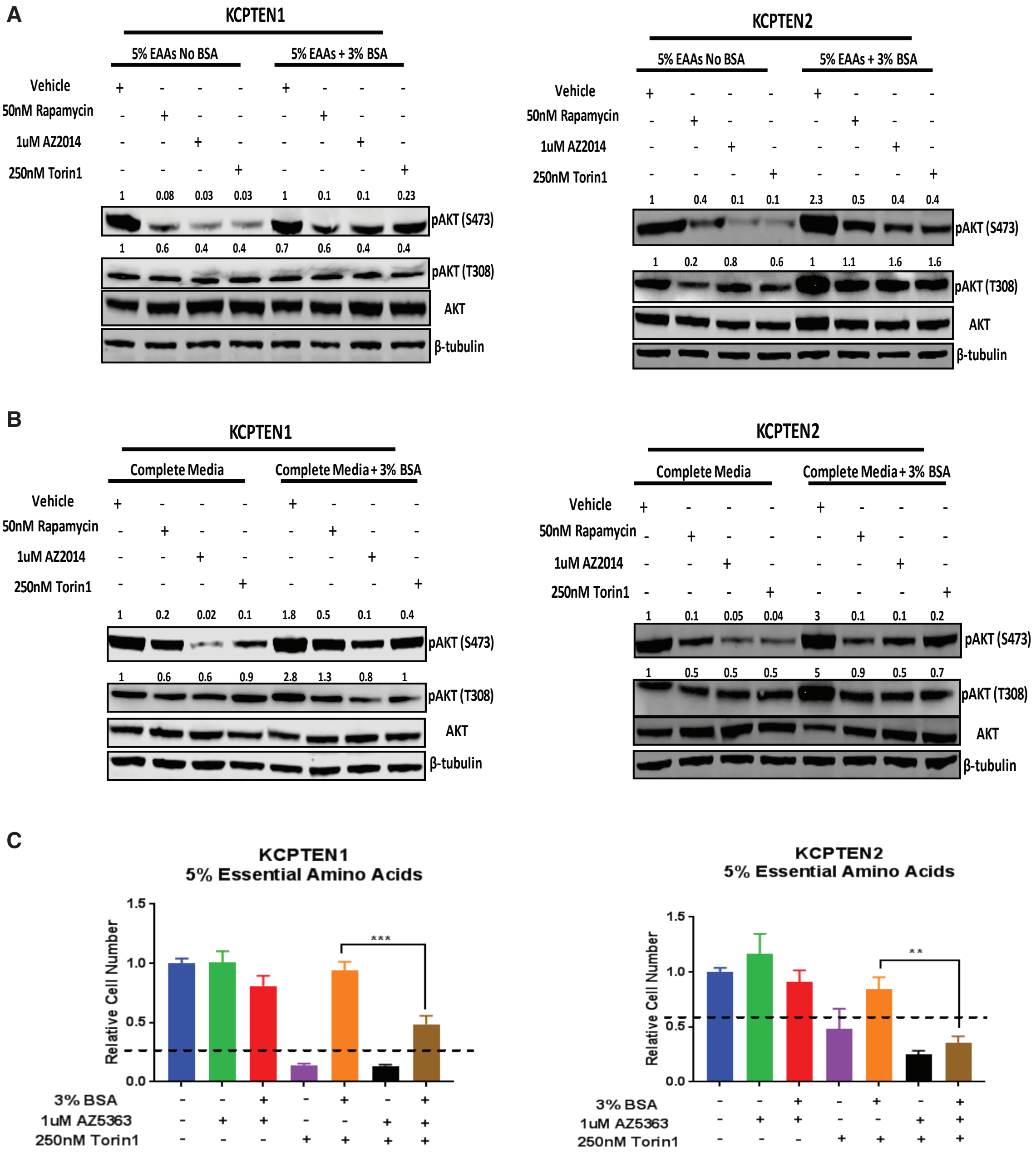

Figure 4. Protein Supplementation Restores AKT Phosphorylation in mTOR-Inhibited KCPTEN Cells

(A) pAKT serine 473 and threonine 308 protein levels in KCPTEN cells cultured in 5% EAA medium. Treatment was with the indicated inhibitors. pAKT (serine 473 and threonine 308) was normalized to the total AKT levels, and each value was expressed relative to the vehicle-treated (no BSA) control. Each blot is representative of at least two independent experiments.

(B) As (A), but in medium with starting DMEM concentrations of EAAs.

(C) Effect of AKT inhibition on proliferation of KCPTEN cells. Cells were cultured in medium with 5% EAAs ±3% BSA, treated with the AKT inhibitor AZD5363 (1 μM) and/or Torin1 (250 nM) for 72 h. Values were normalized to the untreated (no BSA) control.

Error bars represent SEM of three biological experiments, each conducted with three technical replicates. Significance was determined by unpaired t test with Welch's correction. ***$p < 0.0005$ and **$p < 0.001$. Dashed lines indicate the starting cell number.

(Figures S6E and S6F). The abolished proliferation was associated with induction of cell death via apoptosis (Figure S6G). Thus, lysosomal hydrolysis of extracellular protein is critical for the extracellular-protein-mediated resistance against mTOR inhibition.

DISCUSSION

The continuous proliferation of cancer cells puts a high demand on molecular components needed for new cells. Cells meet this

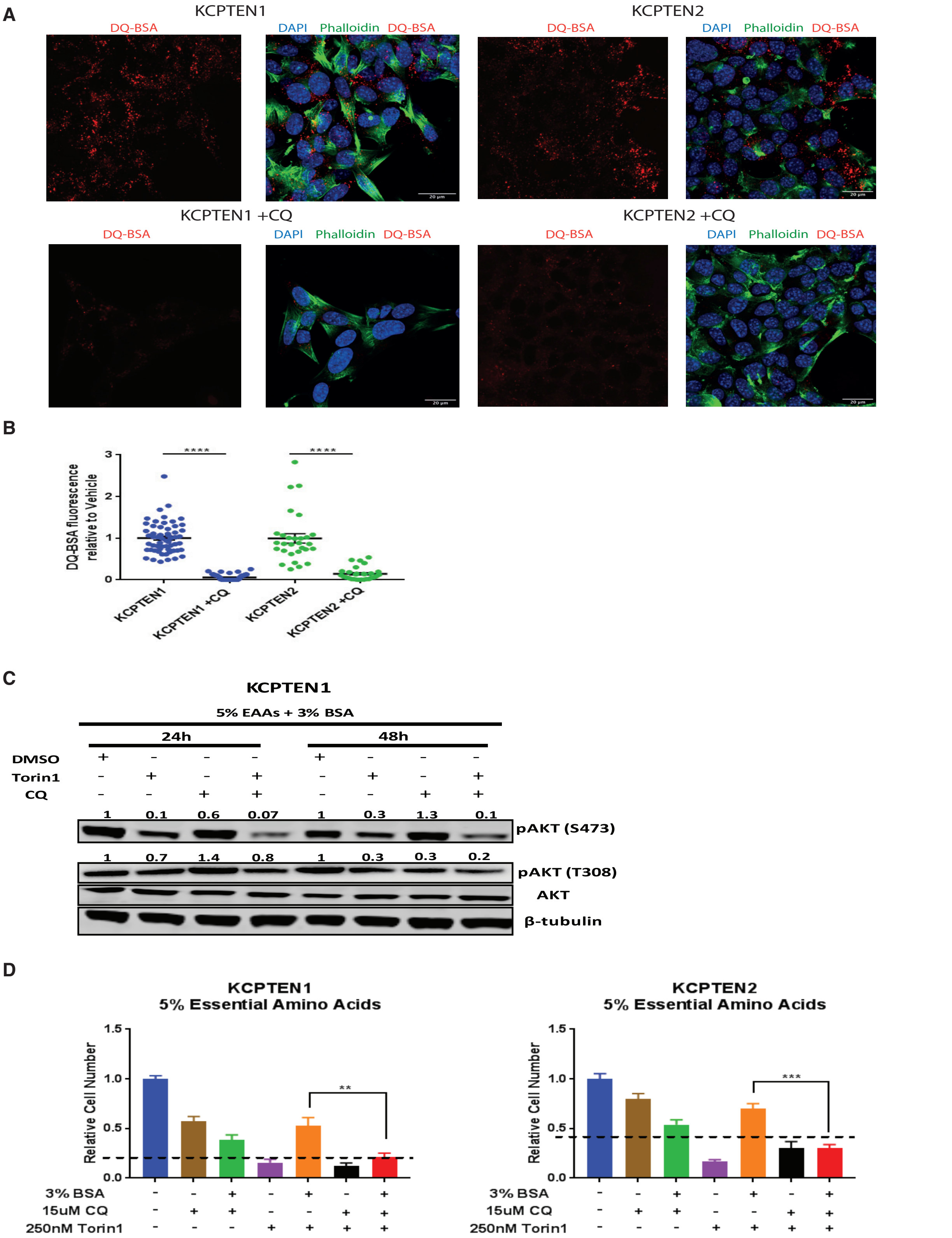

(legend on next page)

by upregulating *de novo* synthetic metabolic pathways (Elstrom et al., 2004; Engelman et al., 2006) or, alternatively, by scavenging and reusing macromolecules (White, 2013). The latter can be critical in a nutrient-restrictive tumor microenvironment, such as in PDAC, where nutrient delivery is poor due to the extensive fibrosis and deficient vasculature (Provenzano et al., 2012; Chauhan et al., 2014; DelGiorno et al., 2014). The decision to commit to *de novo* synthesis or scavenging appears to be, at least in part, governed by oncogenic signaling (Pavlova and Thompson, 2016). Constitutive activation of the PI3K-AKT-mTOR pathway induces nucleotide and fatty acid synthesis, as well as protein translation (Howell et al., 2013), whereas mutant RAS induces both autophagy and macropinocytosis (Commisso et al., 2013; Karsli-Uzunbas et al., 2014). Interestingly, in addition to a near-universal *KRAS* mutation, a significant portion of PDAC tumors also feature loss of *PTEN* (Ying et al., 2011). This is associated with a significantly reduced survival of patients, and mouse models show that when combined, these oncogenic events promote tumor progression (Garcia-Carracedo et al., 2013). What metabolic program do these cells engage in? Our data indicate that both mTOR-mediated anabolic metabolism and macropinocytosis are upregulated in these cells. Critically, our studies with mTOR inhibitors show that the cells can switch between metabolic programs, and this is solely governed by the availability of extracellular protein.

Others have previously established a link between mTOR, specifically mTORC1, and protein scavenging. Palm et al. showed that partial mTOR inhibition could increase cell proliferation in a low-free-EAA but protein-rich environment (Palm et al., 2015). The authors proposed that mTORC1 inhibition relieved its suppressing effect on lysosomal processing of scavenged protein. This was later nuanced by Nofal et al. (2017); it was shown that mTORC1's regulation of lysosomal processing was rather modest and that a more likely explanation for the increased cell proliferation is that partial mTORC1 inhibition balances amino acid demand with supply through macropinocytosis. Some of the results we obtained were subtly different from the work published by these authors. For example, our PDAC-derived KCPTEN cells were not sensitive to a low-EAA environment (i.e., they did not die), as it was observed for MEFs and iBMK cells (Figure S3A; Palm et al., 2015; Nofal et al., 2017). Also, AKT inhibition had opposing effects to what was previously reported. This is most likely caused by the specific cell types used and the genetic alterations studied.

Apart from the above-mentioned subtle differences between this and previous studies, we also encountered fundamentally new observations with respect to the interaction between mTOR and macropinocytosis. First, while both previous reports focused on the interaction between mTORC1 and macropinocytosis, our PDAC-derived cells appear to be mTORC2 rather than mTORC1 driven; in contrast to dual mTOR inhibitors, rapamycin, a mTORC1-specific inhibitor, does not substantially compromise proliferation of KCPTEN (or KPC) cells (Figures S3E and S3F), and hence, there was no proliferative rescue with protein supplementation. Interestingly, rapamycin has been shown to cause proliferative arrest in KCPTEN-bearing mice (Morran et al., 2014). However, it has been shown that prolonged treatment with rapamycin can also lead to mTORC2 inhibition (Sarbassov et al., 2006; Schreiber et al., 2015). We further validated our results by genetically disrupting mTORC1 and mTORC2 via RAPTOR and RICTOR knockout, respectively. Indeed, RAPTOR ablation had no effect on proliferation or macropinocytosis, while it significantly increased lysosomal processing of extracellular protein, as has also been shown by others (Figures S4C–S4E). Interestingly, disruption of mTOR2 complex by knocking out RICTOR abolished protein scavenging, which consequently led to significantly compromised proliferation and induction of apoptosis, which could no longer be recovered by protein supplementation (Figures 3F–3H). The different outcomes of the pharmacological inhibition and the genetic disruption of mTORC2 indicate that beyond its kinase activity, the mTORC2 complex is a critical mediator macropinocytosis and lysosomal functioning (Figure 6). The exact underlying mechanism will be subject to future research. Overall, our data argue that combining lysosomal inhibition with mTOR inhibitors is an efficient therapeutic strategy against PTEN-loss-driven PDAC cells.

The second fundamentally novel observation we made was that protein-mediated rescue of cell proliferation during mTOR inhibition is not due to balancing amino acid supply and demand; rather, it is due to recovery of AKT phosphorylation at serine 473, which is a well-established direct phosphorylation target of mTORC2 (Sarbassov et al., 2005). This at least partially leads to recovery of proliferation, and it suggests that AKT contributes to cell proliferation and hence events such as protein translation independently of mTOR signaling (Figure 6).

How does macropinocytosis lead to recovery of AKT phosphorylation? Our experiments with the lysosomal inhibitor chloroquine (Figure 5C) argue that hydrolysis of the extracellular protein is a step of critical importance for this event to occur.

Figure 5. Macropinocytosis Maintains AKT Phosphorylation, and Its Abrogation Restores Sensitivity to mTOR Inhibition

(A) Lysosomal degradation of extracellular protein was measured using DQ-BSA. KC-PTEN cells were treated with vehicle (DMSO) or chloroquine (CQ; 50 μM) for 4 h. Alexa 488-phalloidin (green) was used for F-actin and DAPI (blue) for nuclear visualization. Scale bar represents 20 μm.

(B) Quantification of DQ-BSA fluorescence upon CQ treatment. An average of 50 z stack images were acquired per condition. Values were normalized to the vehicle-treated (DMSO) control.

(C) KCPTEN1 cells were cultured in medium containing 5% EAAs +3% BSA and treated with Torin1, CQ, and their combination for 24 and 48 h. pAKT (serine 473 and threonine 308) was normalized to the total AKT protein levels and each value was expressed relative to the vehicle-treated (no BSA) control. Blocking lysosomal activity inhibits recovery of pAKT serine 473, bypassing the effect of protein supplementation.

(D) Combinatorial treatment with mTOR and lysosomal inhibition abolishes proliferative advantage conferred by protein. The same cell number was plated, and cells were left overnight to attach. Medium was then changed to 5% EAAs ±3% BSA and cells treated with 30 μM CQ, 250 nM Torin1, or the combination for 72 h. Cell number was used as a readout.

For (B), error bars represent SEM of two independent experiments each conducted in triplicate wells. For (D), error bars represent SEM of three biological experiments, each conducted with three technical replicates. Significance was determined by two-way ANOVA. ****$p < 0.0005$, ***$p < 0.0001$, and **$p < 0.001$.

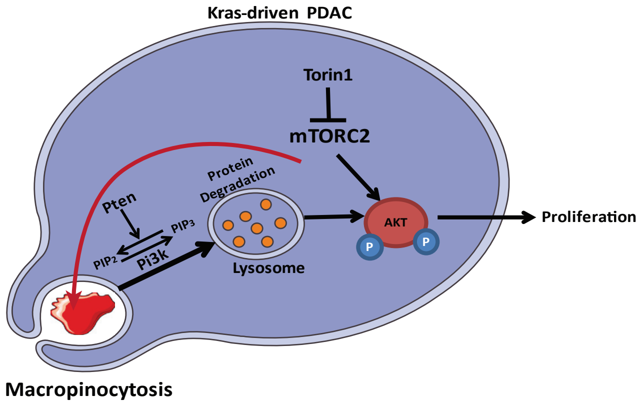

Figure 6. Working Model

Pancreatic cancer cells with concurrent KRAS activation and PTEN loss upregulate the uptake of extracellular protein via macropinocytosis. Both PI3K and AKT play an important role in the regulation of this mechanism. mTORC2 scaffold was found to regulate both uptake (macropinocytosis) and lysosomal processing of scavenged extracellular proteins. Under mTOR-inhibited conditions, lysosomal hydrolysis of extracellular protein leads to recovery of AKT phosphorylation at the serine 473 site, which restores proliferation. Blocking lysosomes with CQ inhibits degradation of scavenged protein and resensitizes PDAC cells with concurrent KRAS activation and PTEN loss to mTOR inhibition.

From this, it is reasonable to propose that protein-derived amino acids play an important role. It is interesting to note that we see protein-mediated rescue of AKT phosphorylation also occurring in high-free-amino-acid conditions. It may be that it is free amino acid concentrations at certain locations in the cell (lysosome versus cytoplasm) that mediate AKT phosphorylation. Alternatively, lysosomal processing of extracellular proteins may activate, in a yet-unknown manner, kinases that would phosphorylate and activate AKT serine 473 when mTORC2 is inhibited. For example, it has been shown that integrin-linked kinase (ILK) and DNA-activated protein kinase (DNA-PK) can bind and phosphorylate the serine 473 site of AKT (Persad et al., 2000; Feng et al., 2004). The exact link between lysosomal hydrolysis and AKT phosphorylation is subject to further interrogation.

Inhibition of mTOR activity (particularly mTORC1) has received significant attention as a therapeutic modality, but clinical results, including in PDAC, have been disappointing (Javle et al., 2010). The fact that mTOR inhibition can promote tumor cell growth in a primary nutrient-poor, but protein-rich, microenvironment has been put forward as an explanation for the lack of clinical success. The results presented here demonstrate that a combination of specific oncogenic alterations and access to extracellular protein is enough to circumvent mTOR inhibition. Protein scavenging is hence a bona fide resistance mechanism. These results should be taken into consideration when investigating the therapeutic effects of mTOR inhibition in the future.

STAR★METHODS

Detailed methods are provided in the online version of this paper and include the following:

- KEY RESOURCES TABLE
- LEAD CONTACT AND MATERIALS AVAILABILITY
- EXPERIMENTAL MODEL AND SUBJECT DETAILS
  - Cell Lines and cell culture conditions
- METHOD DETAILS
  - Essential Amino Acid Starvation
  - RICTOR and RAPTOR knock-out cell lines
  - De novo fatty acid biosynthesis
  - Cell Death Assay (Flow Cytometry)
  - TMR-Dextran and DQ-BSA Assays
  - Western Blotting
- QUANTIFICATION AND STATISTICAL ANALYSIS
- DATA AND CODE AVAILABILITY

SUPPLEMENTAL INFORMATION

Supplemental Information can be found online at https://doi.org/10.1016/j.celrep.2020.01.080.

ACKNOWLEDGMENTS

J.J.K. is supported by a Cancer Research UK Career Development Fellowship (C50242/A17728). We thank the BAIR Imaging facilities at the Beatson Institute for help with imaging and quantification. We also thank Dr. D. Bryant and Dr. A.R. Fernandez for kindly sharing the PTEN plasmid. Finally, we thank the Kamphorst lab and the Cancer Metabolism Research Unit at the Beatson for critical review of the manuscript.

AUTHOR CONTRIBUTIONS

Conceptualization, E.M., O.J.S., and J.J.K.; Methodology, E.M., F.R.A., V.B., A.D.C., S.A.K., J.P.M., and J.J.K.; Investigation, E.M., F.R.A., V.B.; Writing – Original Draft, E.M. and J.J.K.; Writing – Review & Editing, E.M., F.R.A., V.B., A.D.C., S.A.K., J.P.M., O.J.S., and J.J.K.; Funding Acquisition, O.J.S. and J.J.K.; Resources, F.R.A., V.B., A.D.C., S.A.K., J.P.M.; Supervision, O.J.S. and J.J.K.

DECLARATION OF INTERESTS

J.J.K. is an employee and shareholder of Rheos Medicines.

Received: May 19, 2019
Revised: October 14, 2019
Accepted: January 21, 2020
Published: February 25, 2020

REFERENCES

Bar-Sagi, D., and Feramisco, J.R. (1986). Induction of membrane ruffling and fluid-phase pinocytosis in quiescent fibroblasts by ras proteins. Science *233*, 1061–1068.

Bloomfield, G., Traynor, D., Sander, S.P., Veltman, D.M., Pachebat, J.A., and Kay, R.R. (2015). Neurofibromin controls macropinocytosis and phagocytosis in Dictyostelium. eLife *4*, 1–25.

Bryant, K.L., Mancias, J.D., Kimmelman, A.C., and Der, C.J. (2014). KRAS: feeding pancreatic cancer proliferation. Trends Biochem. Sci. *39*, 91–100.

Chauhan, V.P., Boucher, Y., Ferrone, C.R., Roberge, S., Martin, J.D., Stylianopoulos, T., Bardeesy, N., DePinho, R.A., Padera, T.P., Munn, L.L., and Jain, R.K. (2014). Compression of pancreatic tumor blood vessels by hyaluronan is caused by solid stress and not interstitial fluid pressure. Cancer Cell *26*, 14–15.

Commisso, C., Davidson, S.M., Soydaner-Azeloglu, R.G., Parker, S.J., Kamphorst, J.J., Hackett, S., Grabocka, E., Nofal, M., Drebin, J.A., Thompson, C.B., et al. (2013). Macropinocytosis of protein is an amino acid supply route in Ras-transformed cells. Nature *497*, 633–637.

Commisso, C., Flinn, R.J., and Bar-Sagi, D. (2014). Determining the macropinocytic index of cells through a quantitative image-based assay. Nat. Protoc. *9*, 182–192.

Cybulski, N., and Hall, M.N. (2009). TOR complex 2: a signaling pathway of its own. Trends Biochem. Sci. *34*, 620–627.

Davidson, S.M., Jonas, O., Keibler, M.A., Hou, H.W., Luengo, A., Mayers, J.R., Wyckoff, J., Del Rosario, A.M., Whitman, M., Chin, C.R., et al. (2017). Direct evidence for cancer-cell-autonomous extracellular protein catabolism in pancreatic tumors. Nat. Med. *23*, 235–241.

Degenhardt, K., and White, E. (2006). A mouse model system to genetically dissect the molecular mechanisms regulating tumorigenesis. Clin. Cancer Res. *12*, 5298–5304.

Degenhardt, K., Chen, G., Lindsten, T., and White, E. (2002). BAX and BAK mediate p53-independent suppression of tumorigenesis. Cancer Cell *2*, 193–203.

DelGiorno, K.E., Carlson, M.A., Osgood, R., Provenzano, P.P., Brockenbough, J.S., Thompson, C.B., Shepard, H.M., Frost, G.I., Potter, J.D., and Hingorani, S.R. (2014). Response to Chauhan et al.: Interstitial pressure and vascular collapse in pancreas cancer-fluids and solids, measurement and meaning. Cancer Cell *26*, 16–17.

Driscoll, D.R., Karim, S.A., Sano, M., Gay, D.M., Jacob, W., Yu, J., Mizukami, Y., Gopinathan, A., Jodrell, D.I., Evans, T.R., et al. (2016). mTORC2 signaling drives the development and progression of pancreatic cancer. Cancer Res. *76*, 6911–6923.

Dumontier, M., Höcht, P., Mintert, U., and Faix, J. (2000). Rac1 GTPases control filopodia formation, cell motility, endocytosis, cytokinesis and development in Dictyostelium. J. Cell Sci. *113*, 2253–2265.

Ebner, M., Sinkovics, B., Szczygieł, M., Ribeiro, D.W., and Yudushkin, I. (2017). Localization of mTORC2 activity inside cells. J. Cell Biol. *216*, 343–353.

Elstrom, R.L., Bauer, D.E., Buzzai, M., Karnauskas, R., Harris, M.H., Plas, D.R., Zhuang, H., Cinalli, R.M., Alavi, A., Rudin, C.M., and Thompson, C.B. (2004). Akt stimulates aerobic glycolysis in cancer cells. Cancer Res. *64*, 3892–3899.

Engelman, J.A., Luo, J., and Cantley, L.C. (2006). The evolution of phosphatidylinositol 3-kinases as regulators of growth and metabolism. Nat. Rev. Genet. *7*, 606–619.

Feng, J., Park, J., Cron, P., Hess, D., and Hemmings, B.A. (2004). Identification of a PKB/Akt hydrophobic motif Ser-473 kinase as DNA-dependent protein kinase. J. Biol. Chem. *279*, 41189–41196.

Frost, L.S., Dhingra, A., Reyes-Reveles, J., and Boesze-Battaglia, K. (2017). The use of DQ-BSA to monitor the turnover of autophagy-associated cargo. Methods Enzymol. *587*, 43–54.

Garcia-Carracedo, D., Turk, A.T., Fine, S.A., Akhavan, N., Tweel, B.C., Parsons, R., Chabot, J.A., Allendorf, J.D., Genkinger, J.M., Remotti, H.E., and Su, G.H. (2013). Loss of PTEN expression is associated with poor prognosis in patients with intraductal papillary mucinous neoplasms of the pancreas. Clin. Cancer Res. *19*, 6830–6841.

Georgescu, M.M. (2010). Pten tumor suppressor network in PI3K-Akt pathway control. Genes Cancer *1*, 1170–1177.

Guo, J.Y., Chen, H.Y., Mathew, R., Fan, J., Strohecker, A.M., Karsli-Uzunbas, G., Kamphorst, J.J., Chen, G., Lemons, J.M., Karantza, V., et al. (2011). Activated Ras requires autophagy to maintain oxidative metabolism and tumorigenesis. Genes Dev. *25*, 460–470.

Hill, R., Calvopina, J.H., Kim, C., Wang, Y., Dawson, D.W., Donahue, T.R., Dry, S., and Wu, H. (2010). PTEN loss accelerates KrasG12D-induced pancreatic cancer development. Cancer Res. *70*, 7114–7124.

Hingorani, S.R., Wang, L., Multani, A.S., Combs, C., Deramaudt, T.B., Hruban, R.H., Rustgi, A.K., Chang, S., and Tuveson, D.A. (2005). Trp53R172H and KrasG12D cooperate to promote chromosomal instability and widely metastatic pancreatic ductal adenocarcinoma in mice. Cancer Cell *7*, 469–483.

Howell, J.J., Ricoult, S.J., Ben-Sahra, I., and Manning, B.D. (2013). A growing role for mTOR in promoting anabolic metabolism. Biochem. Soc. Trans. *41*, 906–912.

Hruban, R.H., Goggins, M., Parsons, J., and Kern, S.E. (2000). Progression model for pancreatic cancer. Clin. Cancer Res *6*, 2969–2972.

Javle, M.M., Shroff, R.T., Xiong, H., Varadhachary, G.A., Fogelman, D., Reddy, S.A., Davis, D., Zhang, Y., Wolff, R.A., and Abbruzzese, J.L. (2010). Inhibition of the mammalian target of rapamycin (mTOR) in advanced pancreatic cancer: results of two phase II studies. BMC Cancer *10*, 368.

Kamphorst, J.J., Cross, J.R., Fan, J., de Stanchina, E., Mathew, R., White, E.P., Thompson, C.B., and Rabinowitz, J.D. (2013). Hypoxic and Ras-transformed cells support growth by scavenging unsaturated fatty acids from lysophospholipids. Proc. Natl. Acad. Sci. USA *110*, 8882–8887.

Kamphorst, J.J., Nofal, M., Commisso, C., Hackett, S.R., Lu, W., Grabocka, E., Vander Heiden, M.G., Miller, G., Drebin, J.A., Bar-Sagi, D., et al. (2015). Human pancreatic cancer tumors are nutrient poor and tumor cells actively scavenge extracellular protein. Cancer Res. *75*, 544–553.

Karsli-Uzunbas, G., Guo, J.Y., Price, S., Teng, X., Laddha, S.V., Khor, S., Kalaany, N.Y., Jacks, T., Chan, C.S., Rabinowitz, J.D., and White, E. (2014). Autophagy is required for glucose homeostasis and lung tumor maintenance. Cancer Discov. *4*, 914–927.

Kennedy, A.L., Morton, J.P., Manoharan, I., Nelson, D.M., Jamieson, N.B., Pawlikowski, J.S., McBryan, T., Doyle, B., McKay, C., Oien, K.A., et al. (2011). Activation of the PIK3CA/AKT pathway suppresses senescence induced by an activated RAS oncogene to promote tumorigenesis. Mol. Cell *42*, 36–49.

Kim, S.M., Nguyen, T.T., Ravi, A., Kubiniok, P., Finicle, B.T., Jayashankar, V., Malacrida, L., Hou, J., Robertson, J., Gao, D., et al. (2018). PTEN deficiency and AMPK activation promote nutrient scavenging and anabolism in prostate cancer cells. Cancer Discov. 8, 866–883.

Laplante, M., and Sabatini, D.M. (2012). mTOR signaling in growth control and disease. Cell *149*, 274–293.

Lynch, C.J., Fox, H.L., Vary, T.C., Jefferson, L.S., and Kimball, S.R. (2000). Regulation of amino acid-sensitive TOR signaling by leucine analogues in adipocytes. J. Cell. Biochem. *77*, 234–251.

Mann, K.M., Ward, J.M., Yew, C.C., Kovochich, A., Dawson, D.W., Black, M.A., Brett, B.T., Sheetz, T.E., Dupuy, A.J., Chang, D.K., et al.; Australian Pancreatic Cancer Genome Initiative (2012). Sleeping Beauty mutagenesis reveals cooperating mutations and pathways in pancreatic adenocarcinoma. Proc. Natl. Acad. Sci. USA *109*, 5934–5941.

Manning, B.D., and Toker, A. (2017). AKT/PKB signaling: navigating the network. Cell *169*, 381–405.

Mercer, J., and Helenius, A. (2009). Virus entry by macropinocytosis. Nat. Cell Biol. *11*, 510–520.

Michalopoulou, E., Bulusu, V., and Kamphorst, J.J. (2016). Metabolic scavenging by cancer cells: when the going gets tough, the tough keep eating. Br. J. Cancer *115*, 635–640.

Morran, D.C., Wu, J., Jamieson, N.B., Mrowinska, A., Kalna, G., Karim, S.A., Au, A.Y., Scarlett, C.J., Chang, D.K., Pajak, M.Z., et al.; Australian Pancreatic Cancer Genome Initiative (APGI) (2014). Targeting mTOR dependency in pancreatic cancer. Gut *63*, 1481–1489.

Nofal, M., Zhang, K., Han, S., and Rabinowitz, J.D. (2017). mTOR inhibition restores amino acid balance in cells dependent on catabolism of extracellular protein. Mol. Cell *67*, 936–946.e5.

Palm, W., Park, Y., Wright, K., Pavlova, N.N., Tuveson, D.A., and Thompson, C.B. (2015). The utilization of extracellular proteins as nutrients is suppressed by mTORC1. Cell *162*, 259–270.

O'Prey, J., Sakamaki, J., Baudot, A.D., New, M., Van Acker, T., Tooze, S.A., Long, J.S., and Ryan, K.M. (2017). Application of CRISPR/Cas9 to Autophagy Research. Methods Enzymol. *588*, 79–108.

Palm, W., Araki, J., King, B., DeMatteo, R.G., Thompson, C.B., et al. (2017). Critical role for PI3-kinase in regulating the use of proteins as an amino acid source. Proc. Natl. Acad. Sci. U S A *114*, E8628–E8636.

Pavlova, N.N., and Thompson, C.B. (2016). The emerging hallmarks of cancer metabolism. Cell Metab *23*, 27–47.

Perera, R.M., Stoykova, S., Nicolay, B.N., Ross, K.N., Fitamant, J., Boukhali, M., Lengrand, J., Deshpande, V., Selig, M.K., Ferrone, C.R., et al. (2015). Transcriptional control of autophagy-lysosome function drives pancreatic cancer metabolism. Nature *524*, 361–365.

Pérez-Mancera, P.A., Rust, A.G., van der Weyden, L., Kristiansen, G., Li, A., Sarver, A.L., Silverstein, K.A., Grützmann, R., Aust, D., Rümmele, P., et al.; Australian Pancreatic Cancer Genome Initiative (2012). The deubiquitinase USP9X suppresses pancreatic ductal adenocarcinoma. Nature *486*, 266–270.

Persad, S., Attwell, S., Gray, V., Delcommenne, M., Troussard, A., Sanghera, J., and Dedhar, S. (2000). Inhibition of integrin-linked kinase (ILK) suppresses activation of protein kinase B/Akt and induces cell cycle arrest and apoptosis of PTEN-mutant prostate cancer cells. Proc. Natl. Acad. Sci. USA *97*, 3207–3212.

Provenzano, P.P., Cuevas, C., Chang, A.E., Goel, V.K., Von Hoff, D.D., and Hingorani, S.R. (2012). Enzymatic targeting of the stroma ablates physical barriers to treatment of pancreatic ductal adenocarcinoma. Cancer Cell *21*, 418–429.

Rabinowitz, J.D., and White, E. (2010). Autophagy and metabolism. Science *330*, 1344–1348.

Rahib, L., Smith, B.D., Aizenberg, R., Rosenzweig, A.B., Fleshman, J.M., and Matrisian, L.M. (2014). Projecting cancer incidence and deaths to 2030: the unexpected burden of thyroid, liver, and pancreas cancers in the United States. Cancer Res. *74*, 2913–2921.

Recouvreux, M.V., and Commisso, C. (2017). Macropinocytosis: a metabolic adaptation to nutrient stress in cancer. Front. Endocrinol. (Lausanne) *8*, 261.

Rosenfeldt, M.T., O'Prey, J., Flossbach, L., Nixon, C., Morton, J.P., Sansom, O.J., and Ryan, K.M. (2017). PTEN deficiency permits the formation of pancreatic cancer in the absence of autophagy. Cell Death Differ. *24*, 1303–1304.

Sarbassov, D.D., Guertin, D.A., Ali, S.M., and Sabatini, D.M. (2005). Phosphorylation and regulation of Akt/PKB by the rictor-mTOR complex. Science *307*, 1098–1101.

Sarbassov, D.D., Ali, S.M., Sengupta, S., Sheen, J.H., Hsu, P.P., Bagley, A.F., Markhard, A.L., and Sabatini, D.M. (2006). Prolonged rapamycin treatment inhibits mTORC2 assembly and Akt/PKB. Mol. Cell *22*, 159–168.

Scarpa, A., Capelli, P., Mukai, K., Zamboni, G., Oda, T., Iacono, C., and Hirohashi, S. (1993). Pancreatic adenocarcinomas frequently show p53 gene mutations. Am. J. Pathol. *142*, 1534–1543.

Schneider, C.A., Rasband, W.S., and Eliceiri, K.W. (2012). NIH Image to ImageJ: 25 years of image analysis. Nat. Methods *9*, 671–675.

Schönleben, F., Qiu, W., Ciau, N.T., Ho, D.J., Li, X., Allendorf, J.D., Remotti, H.E., and Su, G.H. (2006). PIK3CA mutations in intraductal papillary mucinous neoplasm/carcinoma of the pancreas. Clin. Cancer Res. *12*, 3851–3855.

Schreiber, K.H., Ortiz, D., Academia, E.C., Anies, A.C., Liao, C.Y., and Kennedy, B.K. (2015). Rapamycin-mediated mTORC2 inhibition is determined by the relative expression of FK506-binding proteins. Aging Cell *14*, 265–273.

Tong, X., Zhao, F., and Thompson, C.B. (2009). The molecular determinants of de novo nucleotide biosynthesis in cancer cells. Curr. Opin. Genet. Dev. *19*, 32–37.

Tumanov, S., Bulusu, V., and Kamphorst, J.J. (2015). Analysis of Fatty Acid Metabolism Using Stable Isotope Tracers and Mass Spectrometry. Methods Enzymol. *561*, 197–217.

Vadlakonda, L., Dash, A., Pasupuleti, M., Anil Kumar, K., and Reddanna, P. (2013). The paradox of Akt-mTOR interactions. . Front. Oncol *3*, 165.

Veltman, D.M., Williams, T.D., Bloomfield, G., Chen, B.C., Betzig, E., Insall, R.H., and Kay, R.R. (2016). A plasma membrane template for macropinocytic cups. eLife *5*, , e20085.

White, E. (2013). Exploiting the bad eating habits of Ras-driven cancers. Genes Dev. *27*, 2065–2071.

White, E., and DiPaola, R.S. (2009). The double-edged sword of autophagy modulation in cancer. Clin. Cancer Res. *15*, 5308–5316.

Wiza, C., Nascimento, E.B.M., and Ouwens, D.M. (2012). Role of PRAS40 in Akt and mTOR signaling in health and disease. Am. J. Physiol. Endocrinol. Metab. *302*, E1453–E1460.

Wolpin, B.M., Hezel, A.F., Abrams, T., Blaszkowsky, L.S., Meyerhardt, J.A., Chan, J.A., Enzinger, P.C., Allen, B., Clark, J.W., Ryan, D.P., and Fuchs, C.S. (2009). Oral mTOR inhibitor everolimus in patients with gemcitabine-refractory metastatic pancreatic cancer. J. Clin. Oncol. *27*, 193–198.

Ying, H., Elpek, K.G., Vinjamoori, A., Zimmerman, S.M., Chu, G.C., Yan, H., Fletcher-Sananikone, E., Zhang, H., Liu, Y., Wang, W., et al. (2011). PTEN is a major tumor suppressor in pancreatic ductal adenocarcinoma and regulates an NF-κB-cytokine network. Cancer Discov. *1*, 158–169.

STAR★METHODS

KEY RESOURCES TABLE

REAGENT or RESOURCE	SOURCE	IDENTIFIER
ANTIBODIES		
Rabbit Monoclonal anti-phosphoAKT (Ser473)	Cell Signaling Technology	Cat#4060S; RRID:AB_2315049
Rabbit Monoclonal anti-phosphoAKT (Thr308)	Cell Signaling technology	Cat#4056S; RRID:AB_331163
Rabbit Monoclonal anti-AKT	Cell Signaling Technology	Cat#4685S
Rabbit Monoclonal anti-P70SK	Cell Signaling Technology	Cat#2708S; RRID:AB_390722
Rabbit Monoclonal anti-phosphoP70SK (Thr389)	Cell Signaling Technology	Cat#9234S; RRID:AB_2269803
Rabbit Monoclonal anti-phosphoS6-RP (Ser235/236)	Cell Signaling Technology	Cat#4858S; RRID:AB_916156
Rabbit Monoclonal anti-S6-RP (5G10)	Cell Signaling Technology	Cat#2217S; RRID:AB_331355
Rabbit Monoclonal anti-phosphoPRAS40 (Thr246) (D4D2)	Cell Signaling Technology	Cat#13175S; RRID:AB_2798140
Rabbit Monoclonal anti-PRAS40 (D23C7)	Cell Signaling Technology	Cat#2691; RRID:AB_2225033
Rabbit anti-4EBP1 (53H11)	Cell Signaling Technology	Cat#9644S; RRID:AB_2097841
Rabbit Monoclonal anti-ATG7	Cell Signaling Technology	Cat#8558S; RRID:AB_10831194
Rabbit Monoclonal anti-PTEN	Cell Signaling Technology	Cat#9559S; RRID:AB_390810
Mouse anti-RAPTOR (24C12)	Cell Signaling Technology	Cat#2280S; RRID:AB_561245
Mouse Polyclonal anti-RICTOR	Cell Signaling Technology	Cat#2140; RRID:AB_2179961
Mouse Monoclonal anti-phosphoAKT2 (Ser474) (D3H2)	Cell Signaling Technology	Cat#8599; RRID:AB_2630347
Mouse Monoclonal anti-AKT2	Cell Signaling Technology	Cat#3063S; RRID:AB_2225186
Donkey anti-mouse IRDye 800CW	Fisher Scientific	Cat#926-32212; RRID:AB_621847
Goat anti-Rabbit, Alexa Fluor 488	Invitrogen	Cat# A-11008; RRID:AB_143165
Mouse monoclonal anti-β tubulin	Sigma	Cat#T4026; RRID:AB_477577
CHEMICAL; PEPTIDES; AND RECOMBINANT PROTEINS		
AKT-VIII, AKT inhibitor	Cayman Chemicals	CAY14870
AZ2014, mTORC1/2 inhibitor	AstraZeneca	N/A
AZ5363, AKT inhibitor	AstraZeneca	N/A
Chloroquine, lysosomal inhibitor	Sigma	C6628
EIPA, macropinocytosis inhibitor	Sigma	Ca#1154-25-2
Rapamycin, mTORC1 inhibitor	Sigma	Cat#53123-88-9
Torin1, mTORC1/2 inhibitor	Tocris Bioscience	Cat#4247
Cytochalasin D, macropinocytosis inhibitor	Cayman Chemicals	CAY11330
Bafilomycin A1, lysosomal inhibitor	Tocris Bioscience	Cat#1334
TMR-Dextran, macropinocytosis marker	Thermo-Scientific	Cat#D1818
DQ-BSA, macropinocytosis/lysosomal marker	Molecular Probes	D12051
Phalloidin conjugated to Alexa488	Thermo-Scientific	Cat#A12397
Clarity Max ECL	Bio-Rad	6305
SuperSignal West Femto Maximum Sensitivity Substrate	Thermo Fisher Scientific	34096
Critical Commercial Assays		
FITC-Annexin V, apoptosis marker	Invitrogen	V13242
EXPERIMENTAL MODELS: CELL LINES		
Mouse Cell Lines: Pdx1-Cre; KrasG12D/+; Trp53R172H/+ for KPC1 (C57BL/6J) and KPC2 (mixed background) cell lines	Dr. Jennifer Morton's Laboratory	N/A
Mouse Cell Lines: Pdx1-Cre; KrasG12D/+; Pten-flox for KCPTEN1 and KCPTEN2 cell lines	Dr. Jennifer Morton's Laboratory	N/A

(*Continued on next page*)

Continued		
REAGENT or RESOURCE	SOURCE	IDENTIFIER
OLIGONUCLEOTIDES		
Primers for RICTOR: CACCGCCCGTCAATATGGC GGCGAT	This paper	N/A
Primers for RAPTORT: CACCGCGATCCGTGTCTA CGACAGG	This paper	N/A
Recombinant DNA		
pLNCX1 MyrAKT2	Addgene	#272894
pQCXIH hPTEN	Dr David Bryan't Laboratory	N/A
Software and Algorithms		
ImageJ	Schneider et al., 2012	https://imagej.nih.gov/ij/
Graph Pad Prism 7	GraphPad Software	https://www.graphpad.com/scientific-software/prism/
Image Studio Lite	Li-Cor Biosciences	https://www.licor.com/bio/image-studio-lite/

LEAD CONTACT AND MATERIALS AVAILABILITY

Further information and requests for resources and reagents should be directed to and will be fulfilled by the Lead Contact, Jurre Kamphorst (jurre.kamphorst@glasgow.ac.uk).

This study did not generate new unique reagents.

EXPERIMENTAL MODEL AND SUBJECT DETAILS

Cell Lines and cell culture conditions

Cell lines were originally derived from two separate Pdx1-Cre; KrasG12D/+; Trp53R172H/+ (KPC) mice (both females) (one with mixed background (KPC2) and one with C57BL/6J (KPC1) or from two separate Pdx1-Cre; KrasG12D/+; Ptenflox/+ (KCPTEN) (females) mice. These cell lines were kindly provided by Dr Jennifer Morton's lab and they have been authenticated. Cells were routinely passaged in Dulbecco's Modified Eagle Medium (DMEM, Sigma) with 25 mM glucose and 2 mM L-glutamine, supplemented with 10% (v/v) fetal bovine serum (FBS; Sigma), split at 80% confluence, and were routinely checked for mycoplasma. The cells were incubated at 37°C and 5% CO2.

METHOD DETAILS

Essential Amino Acid Starvation

For essential amino acid (EAA) starvation experiments, the same number of cells (25.000) was plated in full DMEM media and left overnight to adhere. The following day medium was removed, and cells were rinsed once with PBS. Cells were then cultured in Earle's Balanced Salt Solution (EBSS) medium (Thermo Fisher Scientific) supplemented with 5% (1x) MEM Amino Acids and MEM Non-Essential Amino Acids (Sigma), 100x Vitamins, 2 mM Glutamine, 4.5 mM Glucose (for a final concentration of 10mM), 10% dialyzed FBS (Sigma) and 3% Bovine Serum Albumin (Sigma) for 72h unless indicated otherwise. The following inhibitors were added (along with changing medium and left for 72hours) as indicated: 1 μM A2014, 200 nM AZD8186, 200 nM AZD8835, 1 μM AZD5363 (from AstraZeneca); AKT VIII (Cayman Chemical), 20 μM for TMR-Dextran uptake experiments and 10 μM for 72h proliferation assay); Chloroquine (Sigma, 50 μM for DQ-BSA Assay and 15 μM for 72hour-proliferation Assay), 2 μM Cytochalasin (Cayman Chemical), 50 nM Rapamycin (Sigma); 250 nM Torin1 and 1nM Bafilomycin (Tocris Bioscience). Cell number was used as a readout of proliferation and it was determined using the CASY Cell Counter (Roche). 2.5x 10^4 RICTOR-KO and RAPTOR-KO KCPTEN cells were cultured in full DMEM and left overnight to adhere to plastic. The medium was then changed to 5% Essential amino acid in the presence or absence of 3% Bovine Serum Albumin (BSA) and left for 72 hours prior to cell number count by CASY Cell Counter (Roche) or subjected to the Annexin V protocol as described below.

RICTOR and RAPTOR knock-out cell lines

For CRISPR/Cas9 genome editing of the ATG7, RAPTOR and RICTOR genes we used plasmids kindly provided by Kevin Ryan's lab (O'Prey et al., 2017). The v2.o plasmid (O'Prey et al., 2017) was used to clone guide sequences targeting Rictor: CACCGCCCGTCAA TATGGCGGCGAT, and Raptor: CACCGCGATCCGTGTCTACGACAGG. Lentivirus was generated for each plasmid in HEK293T cells and used to infect the relevant cell line. KCPTEN1 cell line was transfected with 5 μg control plasmid (empty vector) or 5 μg plasmid containing either the Atg7, the Rictor or the Raptor sequence. After two rounds of transfection, cells were subjected to selection with

2 μg/ml puromycin for 7 days. Following puromycin selection, the ATG7, RICTOR and RAPTOR knock-out cell lines were confirmed by assessment of Atg7, Rictor, Raptor, pAKT (S473)/total AKT and pP70-S6K/total P70-S6K protein levels via western blotting.

Retrovirus was produced in φinx cells and used to infect the KCPTEN cells. 5 μg of pLNCX1 Myr-AKT2 plasmid (Addgene #27294) was used to express myr-AKT2 to KCPTEN1 cells following the supplier's protocol. After two rounds of transfection, cells were then subjected to 200 μg/ml neomycin selection for 7 days. Similarly, for expression of human PTEN wild-type in KCPTEN1 cells, 5 μg of the pQCXIH plasmid were used. KCPTEN1 cell line was transfected with pQCXIH control plasmid or wild-type hPTEN and after two rounds of transfection it was subjected to 400 μg/ml hygromycin selection for 7 days. Overexpression was assessed by assessing the protein levels of pAKT2 (S474)/total AKT and Pten via western blotting.

De novo fatty acid biosynthesis

Fatty acid labeling was performed as published previously (Tumanov et al., 2015). DMEM supplemented with 10mM U-13C-glucose and 2mM U-13C-glutamine (sigma) and 10% dFBS was used as indicated. After 72h incubation, cells were placed on ice and medium was aspirated, The cells were then washed 2x with ice-cold PBS, quenched with 0.75 mL of 1:1 v/v PBS:Methanol kept at −20°C. After cell scraping, the extraction solvent was transferred to glass tubes, and the total fatty acids was extracted in 0.5 mL chloroform (kept −20°C) and dried under nitrogen gas. Lipids were saponified and methylated with toluene, methanol and methanolic-HCL, and then vortexed and incubated at 100°C for 60 min. Fatty acid methyl esters were extracted with water and hexane and hexane fraction analyzed by GC/MS (Tumanov et al., 2015). Fraction *de novo* synthesized equals 1-fraction M0.

Cell Death Assay (Flow Cytometry)

Apoptosis and necrosis were assayed by flow cytometry. $3x10^5$ cells were seeded on six-well plates for treatment with mTOR/lysosomal inhibitors or $2.5x10^4$ cells on 12-well plates for RAPTOR-KO and RICTOR-KO KCPTEN1 in full DMEM medium and left O/N to attach. Medium was then replaced with 5% EEAs and cells incubated with mTOR/CQ with or without 3% albumin for 72h. Medium was collected, and cells were washed with PBS which was also collected. After trypsinization, cell suspension was added to the collected medium. A suspension containing 10^5 cells was centrifuged at 2000 g for 5 min and supernatant removed. The pellet was re-suspended with in 1X Annexin-binding buffer containing FITC-Annexin V (Apoptosis marker) (Invitrogen). Samples were mixed gently for 15 min at room temperature. Finally, 100 μg/mL of Propidium Iodide (PI-necrosis marker) were added to the solution and samples were processed in Attune NxT Flow Cytometer (Invitrogen).

TMR-Dextran and DQ-BSA Assays

TMR-Dextran Assay

Cells were seeded on glass coverslips in full DMEM for 24h. Cells were then starved for 2h in serum-free DMEM containing 2 mg/mL glutamine, and then medium was replaced with serum-free DMEM containing vehicle or indicated inhibitor and incubated for 1h. Then, cells were incubated in serum-free medium supplemented with 0.5mg/ml TMR-Dextran (Thermo Fisher Scientific) for 30 min. At the end of the incubation, cells were washed five times with 2ml ice-cold PBS and then immediately fixed in 3.7% paraformaldehyde solution for 15 min. Cellular periphery was visualized with Alexa Fluor 488 Phalloidin (for F-actin staining) (Thermo Fisher Scientific, 20 min incubation). For nuclear staining, cells were mounted with 100 μg/mL DAPI (Thermo Fisher Scientific) for 15 min. Coverslips were washed with 2ml PBS 3 times between each step. Z stack images were obtained with an Olympus confocal FV1000 microscope using standard settings. Quantification of the macropinocytic index was done as previously described (Commisso et al., 2014). Mean fluorescence intensity was determined by calculating the integrated signal from randomly chosen fields and normalized to the cell area determined by fluorescently labeled membrane markers (Alexa 488- Phalloidin for actin).

DQ-BSA Fluorescence Assay

After 2h starvation, serum-free DMEM medium was supplemented with 0.1 mg/mL DQ Red BSA (Molecular Probes) and cells incubated for 4h and treated with either vehicle or 50 μM Chloroquine (Sigma). For Figure 3I, Empty Vector KCPTEN cells were cultured in 5% EAAs ± 250nM Torin1 ± 3% BSA for 24 hours prior to the DQ-BSA assay. RICTOR-KO KCPTEN cells were cultured in 5% EAAs without BSA. Cells were rinsed with ice-cold PBS and fixed with 3.7% paraformaldehyde for 15 min. Alexa Fluor-488 Phalloidin was again used for F-actin and DAPI for nuclear visualization as described above and Z stack images were obtained with an Olympus confocal FV1000 microscope using standard settings. Quantification was carried out as described above.

Western Blotting

Cells were plated at full DMEM medium on 6-cm dishes and left overnight to adhere. Medium was changed as previously described and cells were treated with the indicated concentrations of small-molecule inhibitors. Plates were placed on ice and cells were washed 3x with ice-cold PBS. Whole-cell protein lysates were prepared in RIPA buffer (Thermo Fisher Scientific), containing 100x Protease and Phosphatase inhibitors (Sigma), and total protein concentration was determined by DC protein assay (Bio-Rad). Proteins were separated using precast 4%–12% or 3%–8% NuPAGE gels (Invitrogen, Life Technologies), and transferred onto nitrocellulose membrane. Primary antibodies were used at 1:1000 dilution. Secondary antibodies were IRDye 800CW Donkey anti-Mouse (Thermo Fisher Scientific), or Alexa Fluor 488 Goat anti-Rabbit (Invitrogen), all at 1:5000 dilution. Mouse monoclonal Anti-β-tubulin

(Sigma) at 1:5000 was used as loading control. Proteins were detected and quantified using a Li-Cor Odyssey Infrared scanner and software (Li-Cor Biosciences). ECL was used for the development of membranes incubated with pAKT T308 antibody (Cell Signaling, Cat#4056S).

Quantification of western blots was done using the Image Studio Lite.

QUANTIFICATION AND STATISTICAL ANALYSIS

Graphs were created, and statistical analysis was performed using GraphPad Prism 7.02 software. Statistical details can be found in the figure legends.

DATA AND CODE AVAILABILITY

This study did not generate datasets.

Cell Reports, Volume *30*

Supplemental Information

Macropinocytosis Renders a Subset of Pancreatic

Tumor Cells Resistant to mTOR Inhibition

Evdokia Michalopoulou, Francesca R. Auciello, Vinay Bulusu, David Strachan, Andrew D. Campbell, Jacqueline Tait-Mulder, Saadia A. Karim, Jennifer P. Morton, Owen J. Sansom, and Jurre J. Kamphorst

S1.A

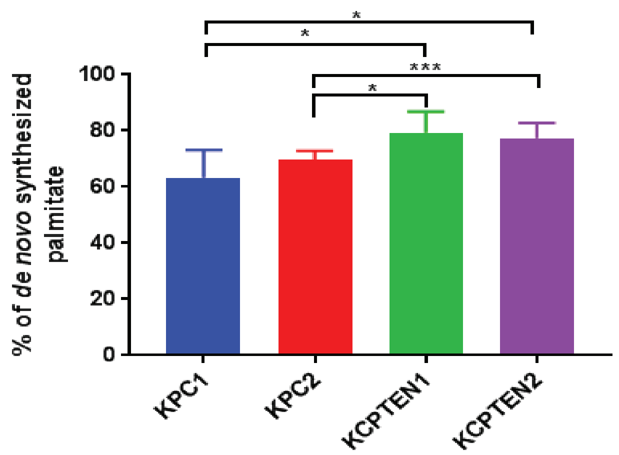

% of de novo synthesized palmitate
100
80
60
40
20
0
*
*
***
*
KPC1
KPC2
KCPTEN1
KCPTEN2

S1.B

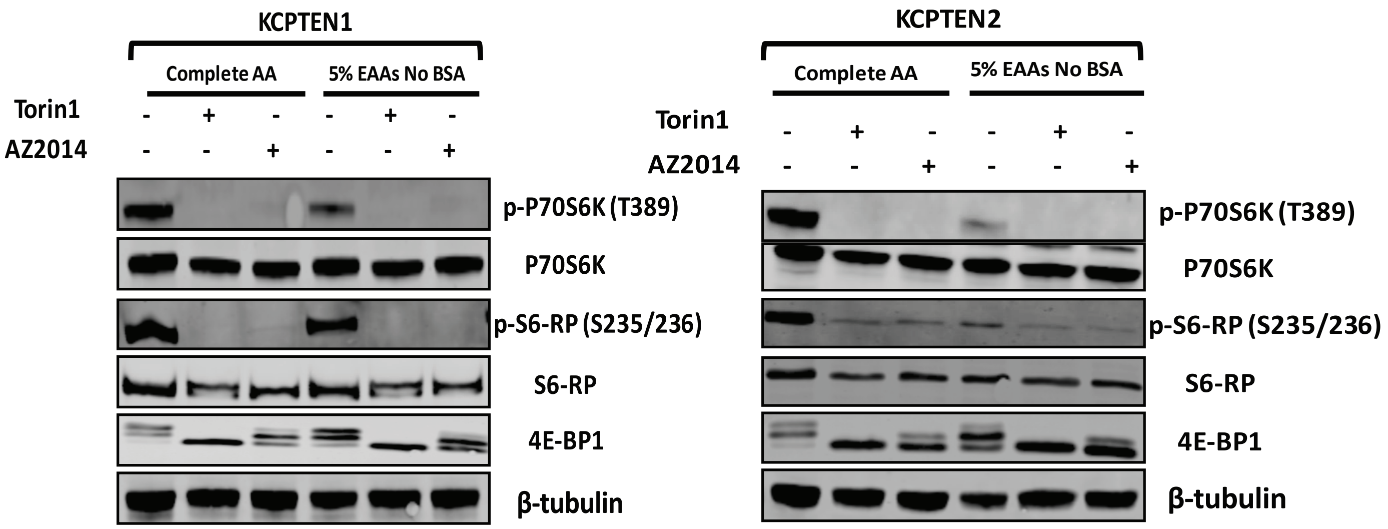

KCPTEN1
Complete AA
5% EAAs No BSA
Torin1
AZ2014
p-P70S6K (T389)
P70S6K
p-S6-RP (S235/236)
S6-RP
4E-BP1
β-tubulin
KCPTEN2
Complete AA
5% EAAs No BSA
Torin1
AZ2014
p-P70S6K (T389)
P70S6K
p-S6-RP (S235/236)
S6-RP
4E-BP1
β-tubulin

S1.C

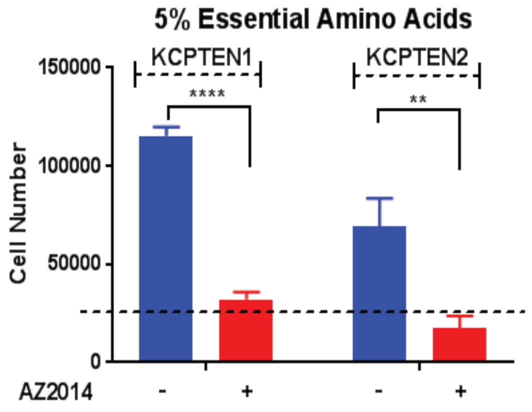

5% Essential Amino Acids
KCPTEN1
KCPTEN2
****
**
Cell Number
150000
100000
50000
0
AZ2014
-
+
-
+

Figure S1. Relate to Figure 1.

KCPTEN cells are sensitive to mTOR inhibition.

- **A** *De novo* synthesized palmitate in KPCs and KCPTENs. Cells were cultured in complete media containing [$U^{13}C$]-glucose and [$U^{13}C$]-glutamine for 72 h.
- **B** mTORC1 signaling activity upon mTOR dual inhibition. Cells were cultured in indicated conditions for 24 h and protein levels of mTORC1 targets were assessed by WB.
- **C** Effect of mTOR dual inhibition (1 µM AZD2014) on proliferation of KC-PTEN cells. Cells were cultured in 5% of DMEM essential amino acid (EAA) concentrations for 72 h.

  For **A**, error bars represent Standard Deviation (SD) of 2 independent experiments each conducted in triplicates and significance was determined by unpaired t-test; for **C**, error bars represent s.e.m. of 3 biological experiments each conducted with 3 technical replicates and significance was determined by Tukey-corrected two-way ANOVA. ****p<0.0001, ***p<0.001, *p<0.01

Dashed line indicates the starting cell number.

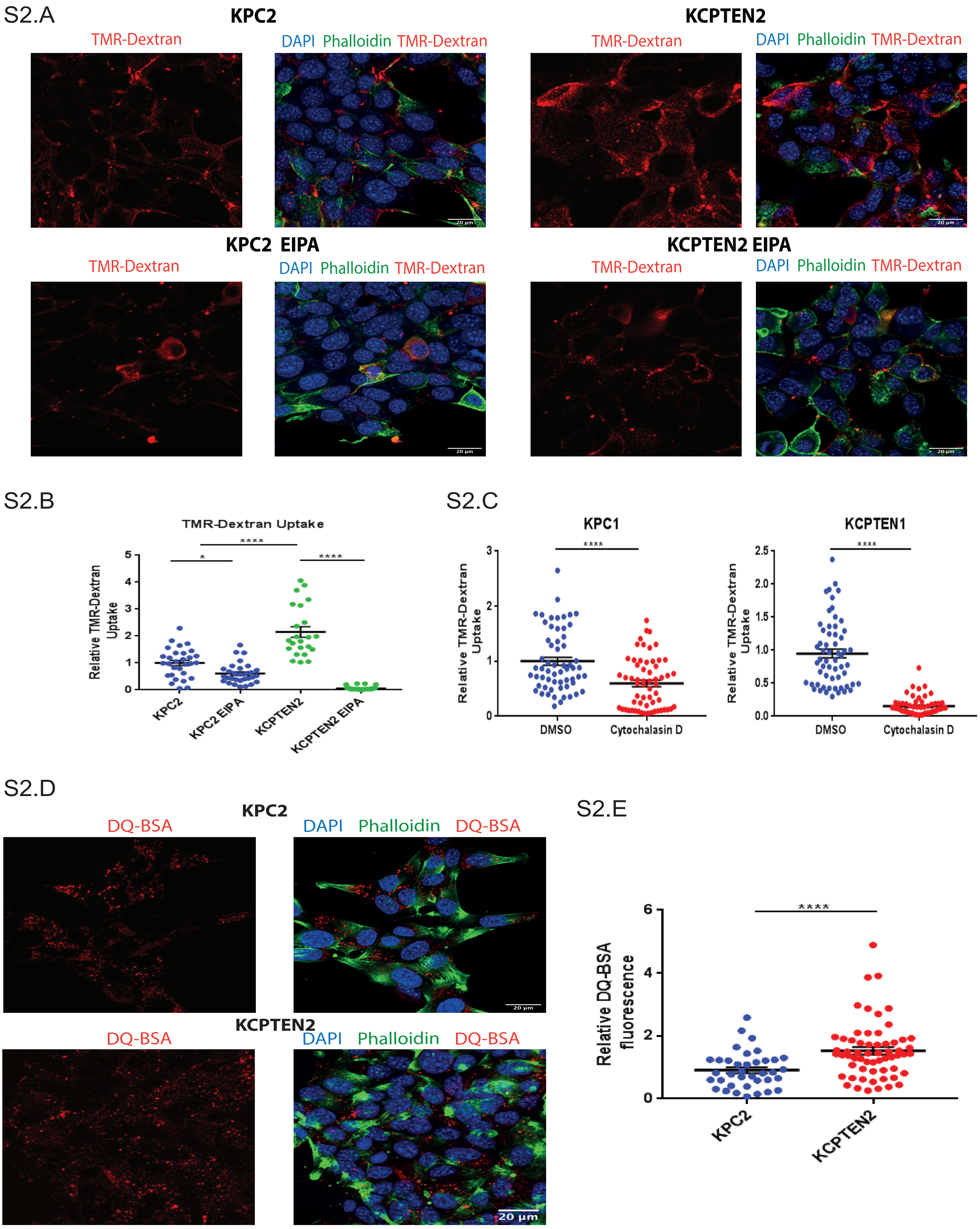
S2.A
KPC2
TMR-Dextran
DAPI Phalloidin TMR-Dextran
KCPTEN2
TMR-Dextran
DAPI Phalloidin TMR-Dextran
KPC2 EIPA
TMR-Dextran
DAPI Phalloidin TMR-Dextran
KCPTEN2 EIPA
TMR-Dextran
DAPI Phalloidin TMR-Dextran
S2.B
TMR-Dextran Uptake
Relative TMR-Dextran Uptake
0
1
2
3
4
5
*
****
****
KPC2
KPC2 EIPA
KCPTEN2
KCPTEN2 EIPA
S2.C
KPC1
Relative TMR-Dextran Uptake
0
1
2
3
****
DMSO
Cytochalasin D
KCPTEN1
Relative TMR-Dextran Uptake
0.0
0.5
1.0
1.5
2.0
2.5
****
DMSO
Cytochalasin D
S2.D
KPC2
DQ-BSA
DAPI Phalloidin DQ-BSA
20 µm
KCPTEN2
DQ-BSA
DAPI Phalloidin DQ-BSA
20 µm
S2.E
Relative DQ-BSA fluorescence
0
2
4
6
****
KPC2
KCPTEN2

Figure S2. Related to Figure 2.

KCPTEN cells exhibit increased uptake and processing of extracellular material via macropinocytosis.

- **A** Macropinocytosis assay using TMR-Dextran as a marker of macropinosomes (red staining) in KPC and KCPTEN cells. Nuclei stained with DAPI (blue) (49,6-diamidino-2-Phenylindole) and Alexa-488 Phalloidin (green) for actin staining (cellular periphery). The amiloride macropinocytosis inhibitor EIPA was used at 50 µM (1 hour).
- **B** Quantification of TMR-Dextran fluorescence. An average of 30 images was acquired per condition. Values were normalized to the average of untreated KPC2 (DMSO).
- **C** Same as **B**, but 2 µM Cytochalasin D (1 hour) was used to block macropinocytosis.
- **D** Lysosomal processing of extracellular proteins up-taken via macropinocytosis was assessed by DQ-BSA fluorescence.
- **E** Quantification of DQ-BSA fluorescence. An average of 40 z-stack images was acquired per cell line. Values were normalized to the average of KPC2.

  For **B, C** & **D**, error bar represents s.e.m. of 2 biological experiments each with 3 technical replicates. For **B** & **D**, significance was determined by one-way ANOVA. For **C,** significance was determined by unpaired student's t-test with Welch's corrections. ns, non-significant. ****$p<0.0001$, *$p<0.05$.

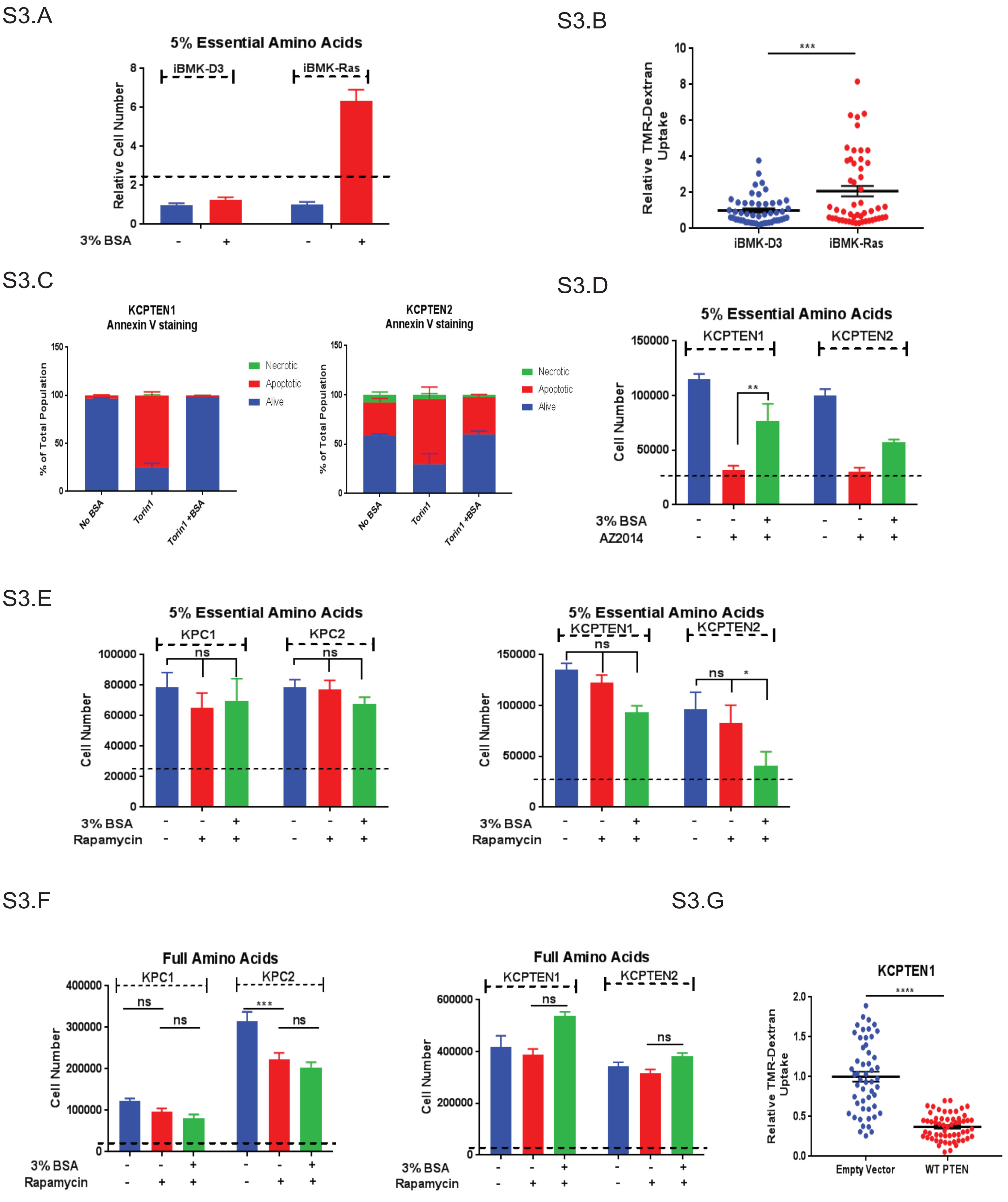
S3.A
5% Essential Amino Acids
iBMK-D3
iBMK-Ras
Relative Cell Number
3% BSA
S3.B
***
Relative TMR-Dextran Uptake
iBMK-D3
iBMK-Ras
S3.C
KCPTEN1
Annexin V staining
KCPTEN2
Annexin V staining
% of Total Population
Necrotic
Apoptotic
Alive
No BSA
Torin1
Torin1 +BSA
S3.D
5% Essential Amino Acids
KCPTEN1
KCPTEN2
**
Cell Number
3% BSA
AZ2014
S3.E
5% Essential Amino Acids
KPC1
KPC2
ns
Cell Number
3% BSA
Rapamycin
5% Essential Amino Acids
KCPTEN1
KCPTEN2
ns
*
Cell Number
3% BSA
Rapamycin
S3.F
Full Amino Acids
KPC1
KPC2
ns
***
Cell Number
3% BSA
Rapamycin
Full Amino Acids
KCPTEN1
KCPTEN2
ns
Cell Number
3% BSA
Rapamycin
S3.G
KCPTEN1
****
Relative TMR-Dextran Uptake
Empty Vector
WT PTEN

Figure S3. Related to Figure 3.

Protein scavenging confers resistance against mTORC2 inhibition.

A Effect of BSA supplementation on cell proliferation of iBMK-D3 (control) and iBMK-RAS. Cell number was used as a readout for proliferation. Cells were cultured in medium containing 5% EAAs for 72 h.

B Induction of macropinocytosis by RAS activation measured by TMR-Dextran Uptake in iBMK-Ras cells.

C Effect of Torin1 on cell Death was assessed by Annexin V staining. KCPTEN1 and KCPTEN2 cells were cultured in medium containing 5% EAAs ± 3% BSA for 72hours. [Alive: Annexin/PI negative, Apoptotic: Annexin (+)/PI (-) and Necrotic: Annexin V(+)/PI(+)].

D KCPTEN1 and KCPTEN2 cells cultured in 5% EAAs, treated with the dual mTOR inhibitor AZD2014 (1 µM) ± 3% BSA for 72 h. Cell number was used as a readout of proliferation.

E Proliferation assay for KPCs and KCPTENs in 5% EAAs medium treated with 50 nM Rapamycin ± 3% BSA for 72 h.

F Same as **E**, but cells were cultured in full DMEM with standard amino acid concentrations.

G Quantification of TMR-Dextran fluorescence of E. Vector- and WT hPTEN-expressing KCPTEN1 cells. An average of 60 images was acquired per cell line. Values were expressed as relative to the E. Vector control.

For **A** & **B**, error bars represent s.e.m. of 2 biological experiments each conducted with 3 technical replicates. For **D, E & F**, error bars represent s.e.m of 3 biological experiments and significance was determined by two-way ANOVA with Tukey corrections. For **G** error bars represent s.e.m of 3 independent experiments. For TMR-Dextran Assays (**B** & **G**) an average of 50 z-stack images were acquired, and significance was determined by unpaired student's t-test with Welch corrections. ns for non-significant. ****$p<0.0001$, ***$p<0.005$, **$p<0.05$.

Dashed lines indicate the starting cell number.

S4.A

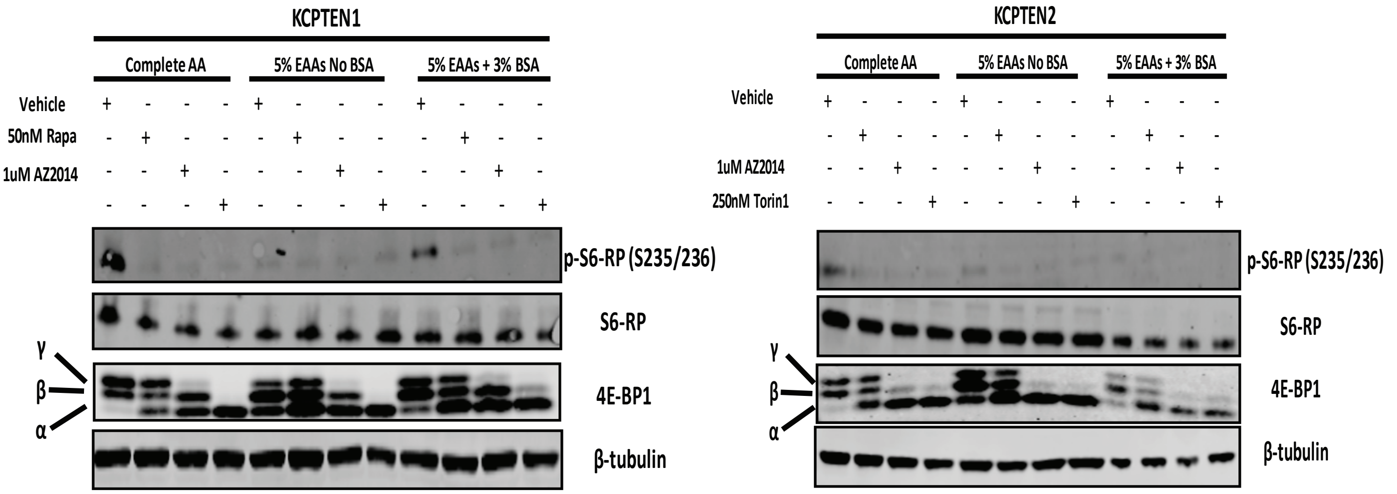

S4.B

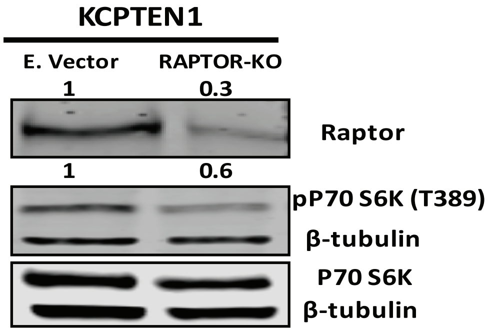

S4.C

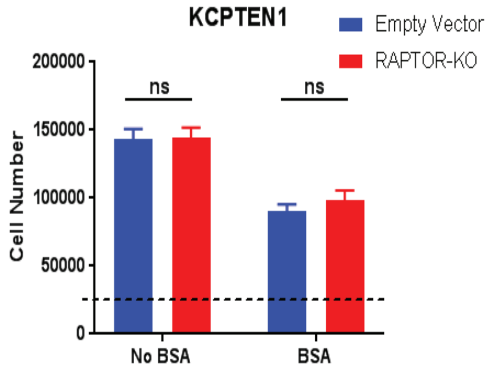

S4.D

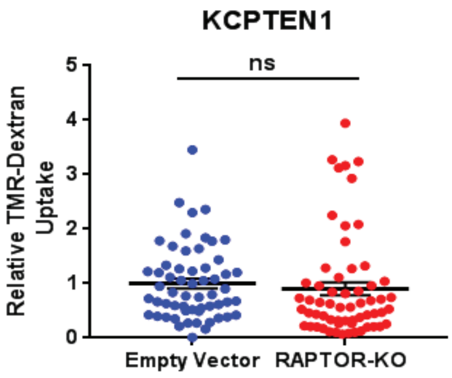

S4.E

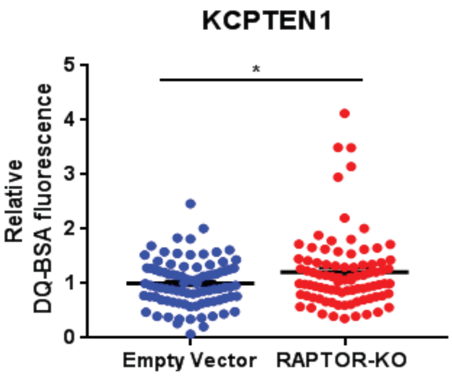

Figure S4. Related to Figure 4.

mTORC1 is not regulating protein scavenging in KCPTEN cells.

- **A** mTORC1 signaling activity upon BSA supplementation and dual mTOR inhibition. Cells were cultured in indicated conditions for 24h and protein levels of mTORC1 downstream targets (pS6-RP, S6-RP and 4E-BP1) were assessed by western blot.
- **B** Raptor, pP70S6K and total P70S6K protein levels in E. Vector- and RAPTOR-KO KCPTEN1 cells. Levels of Raptor were normalized to β-tubulin and values expressed as relative to E. Vector. pP70 S6K was normalized to total P70 S6K and expressed as relative to E. Vector.
- **C** Proliferation assay for RAPTOR-KO KCPTEN1 cells cultured at 5% EAAs ± 3% BSA for 72 hours.
- **D** Quantification of TMR-Dextran uptake and DQ-BSA fluorescence for RAPTOR-KO KCPTEN1 cells. An average of 60 z-stack images was acquired for TMR-Dextran uptake and 90 images for DQ-BSA per cell line. Values were normalized to the average of the Empty Vector-expressing KCPTEN1.

For **C & D** error bars represent s.e.m. of 3 independent experiments. Each biological experiment was performed with 3 technical replicates. Significance was determined by unpaired t-test with Welch's corrections. ns for non-significant, *p<0.01

Dashed lines indicate the starting cell number.

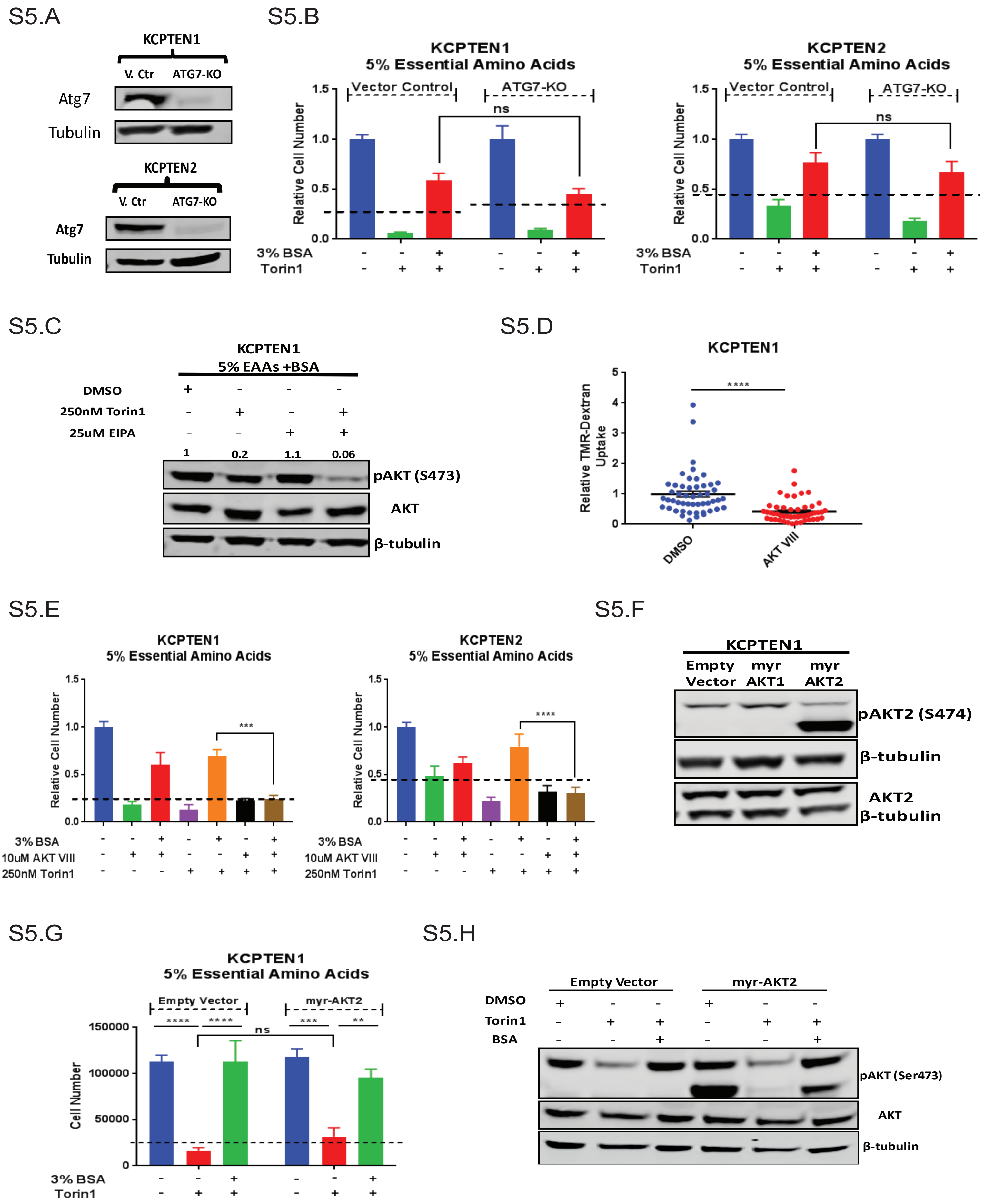
S5.A
KCPTEN1
V. Ctr
ATG7-KO
Atg7
Tubulin
KCPTEN2
V. Ctr
ATG7-KO
Atg7
Tubulin
S5.B
KCPTEN1
5% Essential Amino Acids
Vector Control
ATG7-KO
ns
Relative Cell Number
1.5
1.0
0.5
0.0
3% BSA
Torin1
KCPTEN2
5% Essential Amino Acids
Vector Control
ATG7-KO
ns
Relative Cell Number
3% BSA
Torin1
S5.C
KCPTEN1
5% EAAs +BSA
DMSO
250nM Torin1
25uM EIPA
1
0.2
1.1
0.06
pAKT (S473)
AKT
β-tubulin
S5.D
KCPTEN1
****
Relative TMR-Dextran Uptake
DMSO
AKT VIII
S5.E
KCPTEN1
5% Essential Amino Acids
***
Relative Cell Number
3% BSA
10uM AKT VIII
250nM Torin1
KCPTEN2
5% Essential Amino Acids
****
Relative Cell Number
3% BSA
10uM AKT VIII
250nM Torin1
S5.F
KCPTEN1
Empty Vector
myr AKT1
myr AKT2
pAKT2 (S474)
β-tubulin
AKT2
β-tubulin
S5.G
KCPTEN1
5% Essential Amino Acids
Empty Vector
myr-AKT2
****
****
ns
***
**
Cell Number
150000
100000
50000
0
3% BSA
Torin1
S5.H
Empty Vector
myr-AKT2
DMSO
Torin1
BSA
pAKT (Ser473)
AKT
β-tubulin

Figure S5. Related to Figure 4.

Protein scavenging in PTEN loss-driven PDAC cells is AKT-dependent.

A Western Blot for ATG7 protein levels in KCPTEN1 and KCPTEN2 ATG7-KO cells.

B Proliferation assay for ATG7-KO KCPTEN1 and KC-PTEN2 cells. Cells cultured at 5% of DMEM essential amino acid (EAA) concentrations, treated with 250 nM Torin1 and supplemented with 3% BSA for 72 h.

C pAKT Ser473 and total AKT protein levels in KCPTEN1 cells treated with 25 µM EIPA ± 250 nM Torin1 for 48 hours. pAKT was normalized to total AKT levels and values are expressed relative to DMSO-treated control.

D Quantification of TMR-Dextran uptake for KCPTEN1 cells treated with 20µM of AKT VIII inhibitor for 1 hour. An average of 60 z-stack images was acquired per condition. Values were normalized to the DMSO-treated control.

E Effect of AKT and mTOR inhibition on proliferation of KCPTEN1 and KCPTEN2 cells. Cells were cultured in medium containing 5% EAAs ± 3% BSA and cells treated with 10 µM AKT VIII, 250nM Torin1 and the AKT VIII/Torin1 combination for 72 h.

F pAKT2 (Ser474) and total AKT2 protein levels in Empty Vector- and myrAKT2-expressing KCPTEN1 cells.

G Proliferation assay for KCPTEN1 cells expressing either E. Vector or myrAKT2 cultured in medium containing 5% EAAs ± 3% BSA, treated with 250nM Torin1 for 72 hours.

H pAKT (S473) and total AKT protein levels of KCPTEN1 E. Vector and myrAKT2 cells cultured at the same conditions as (**F**) for 24 hours. The upper band shows phosphorylation of AKT at Serine473 and the lower band indicates the phosphorylated AKT induced by the expression of the myristoylated sequence.

For **B, D & E,** error bars represent s.e.m of 3 biological experiments each conducted with 3 technical replicates. For **B**, significance was determined by unpaired student's t-test with Welch's corrections and for **E & G** significance was determined by Tukey-corrected two-way ANOVA. n-s for non-significant, ****$p<0.0001$, ***$p<0.005$, **$p<0.05$.

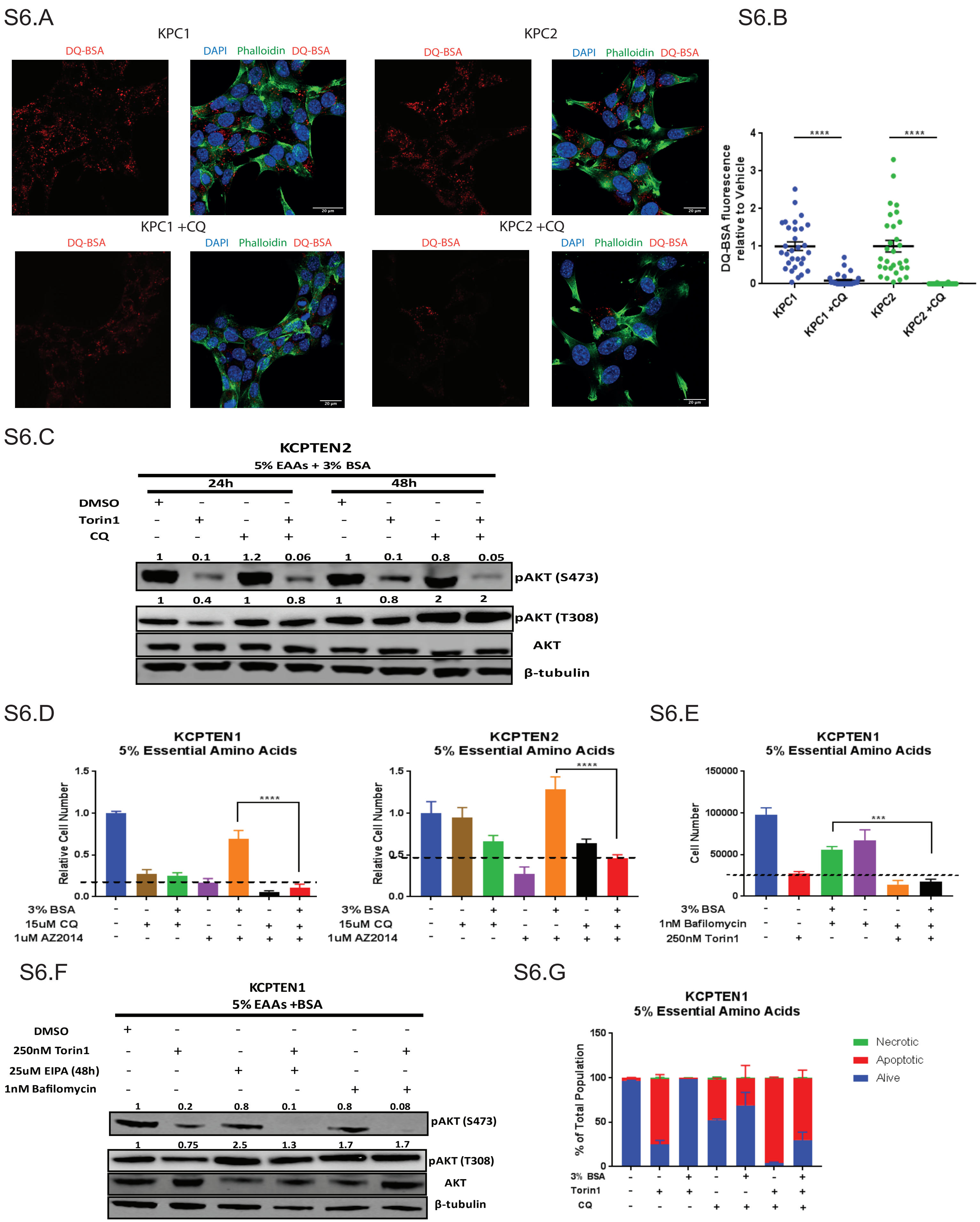
S6.A
KPC1
DQ-BSA
DAPI Phalloidin DQ-BSA
KPC2
DQ-BSA
DAPI Phalloidin DQ-BSA
KPC1 +CQ
DQ-BSA
DAPI Phalloidin DQ-BSA
KPC2 +CQ
DQ-BSA
DAPI Phalloidin DQ-BSA
S6.B
DQ-BSA fluorescence relative to Vehicle
****
****
KPC1
KPC1 +CQ
KPC2
KPC2 +CQ
S6.C
KCPTEN2
5% EAAs + 3% BSA
24h
48h
DMSO
Torin1
CQ
1 0.1 1.2 0.06 1 0.1 0.8 0.05
pAKT (S473)
1 0.4 1 0.8 1 0.8 2 2
pAKT (T308)
AKT
β-tubulin
S6.D
KCPTEN1
5% Essential Amino Acids
Relative Cell Number
****
3% BSA
15uM CQ
1uM AZ2014
KCPTEN2
5% Essential Amino Acids
Relative Cell Number
****
3% BSA
15uM CQ
1uM AZ2014
S6.E
KCPTEN1
5% Essential Amino Acids
Cell Number
***
3% BSA
1nM Bafilomycin
250nM Torin1
S6.F
KCPTEN1
5% EAAs +BSA
DMSO
250nM Torin1
25uM EIPA (48h)
1nM Bafilomycin
1 0.2 0.8 0.1 0.8 0.08
pAKT (S473)
1 0.75 2.5 1.3 1.7 1.7
pAKT (T308)
AKT
β-tubulin
S6.G
KCPTEN1
5% Essential Amino Acids
% of Total Population
Necrotic
Apoptotic
Alive
3% BSA
Torin1
CQ

Figure S6. Related to Figure 5. Combined lysosomal and mTOR inhibition alleviates the macropinocytosis-mediated resistance.

A Intracellular degradation of DQ-BSA in KPC1 and KPC2 cells treated with 50 µM Chloroquine (CQ) for 4 h. Alexa 488-Phalloidin was used for F-actin and DAPI for nuclear visualization. Lysosomal inhibition blocks the degradation of extracellular protein.

B Quantification of DQ-BSA fluorescence. An average of 30 z-stack images was acquired per condition. Values were normalized to the DMSO-treated control.

C KCPTEN2 cells were cultured in medium containing 5% EAAs +3% BSA and treated with Torin1, CQ and the combination for 24 and 48. pAKT Ser473 and Thr308 protein levels were assessed by WB. pAKT (S473 and T308) was normalized to the total AKT levels and each value was expressed relative to the vehicle-treated (No BSA) control. Blocking lysosomal activity inhibits recovery of pAKT S473 bypassing the effect of protein supplementation.

D Effect of lysosomal inhibition on proliferation of mTOR-treated KCPTEN1 and KCPTEN2 cells. Cells were cultured in medium containing 5% EAAs ± 3% BSA and cells treated with 15 µM CQ, 1 µM AZD2014 and the CQ/AZD2014 combination for 72 h.

E Same as **D**, but cells were treated with 1 nM Bafilomycin and 250nM Torin1.

F KCPTEN1 cells cultured in medium containing 5% EAAs ± 3% BSA and treated with 25 µM EIPA, 1nM Bafilomycin and the combination for 48 hours. pAKT Ser473 and Thr308 protein levels were assessed by WB. pAKT (S473 & T308) levels were normalized to total AKT and expressed as relative to DMSO-treated control.

G Cell Death was assessed by Annexin V staining. KCPTEN1 cells were cultured in 5% EAAs ± 3% BSA; treated with 250nM Torin1 and 15µM CQ for 72 hours. [Alive: Annexin/PI negative, Apoptotic: Annexin (+)/PI (-) and Necrotic: Annexin V(+)/PI(+)].

For **B**, error bars represent s.e.m for 2 biological experiments each conducted with 3 technical replicates; for **D**, error bars represent s.e.m. of 3 biological experiments; for **G**, a representative experiment is shown, and error bars represent standard deviation (SD). Cell number was normalized to the vehicle-treated (No BSA) control. Scale bar is 10 µm. For **B,** significance was determined by unpaired student's t-test with Welch's corrections and for **D-E,** it was determined by Tukey-corrected two-way ANOVA. ****p<0.0001, ***p<0.005. Dashed lines indicate the starting cell number.
